# Proteomic profiling of equine airway mucus reveals compositional changes in asthmatic phenotypes

**DOI:** 10.1038/s41598-026-38766-3

**Published:** 2026-02-10

**Authors:** Florian Bartenschlager, Benno Kuropka, Philip Schmitz, Fiona Dumke, Katharina Landmann, Achim D. Gruber, Christoph Weise, Christiane L. Schnabel, Heidrun Gehlen, Lars Mundhenk

**Affiliations:** 1 Institute of Veterinary Pathology, Faculty of Veterinary Medicine, Freie Universität Berlin, Berlin, Germany; 2 Institute of Chemistry and Biochemistry, Department of Biology, Chemistry, Pharmacy , Freie Universität Berlin, Berlin, Germany; 3 Institute of Immunology, Faculty of Veterinary Medicine, Leipzig University, Leipzig, Germany; 4 Equine Clinic, Faculty of Veterinary Medicine, Freie Universität Berlin, Berlin, Germany

**Keywords:** RAO, COB, IAD, Heaves, MUC4, Horses, Biochemistry, Biomarkers, Diseases, Physiology

## Abstract

Mucus hypersecretion and accumulation are hallmark features of equine asthma (EA), a meaningful respiratory disorder in horses occurring in mild to moderate (MEA) and severe (SEA) forms. Changes of the proteomic composition of airway mucus in EA are poorly understood. Using label-free quantitative liquid chromatography-mass spectrometry, we analyzed airway mucus from SEA (*n* = 10), MEA (*n* = 6), and healthy (*n* = 8) horses. We identified and quantified 2,275 proteins including gel-forming mucins MUC5AC and MUC5B and membrane-bound mucins MUC1 and MUC4. Compared with healthy controls, 130 proteins (SEA) and 103 (MEA) were significantly increased. 38 were elevated in SEA relative to MEA, 10 were higher in MEA. MUC4 was markedly increased in both, correlated with bronchoalveolar lavage neutrophils (ρ = 0.790, *p* = 4.9E-06), and distinguished excellently between healthy and asthmatics (AUC = 1.0, 95% CI: 1–1), similar to 23 other proteins. MUC5AC was elevated in both, whereas MUC5B only in SEA. MUC1 did not differ between groups. Changes in mucus-modifying proteins, including glycosyltransferases and aquaporins, suggest altered mucus properties in EA. Functional enrichment analyses revealed inflammation-, tissue remodeling- and coagulation-linked GO terms and pathways in EA. The distinct proteomic profiles add to the understanding of EA and may offer novel targets for phenotype-specific biomarkers and therapy.

**Supplementary Information:**

The online version contains supplementary material available at 10.1038/s41598-026-38766-3.

## Introduction

Equine asthma (EA) is the most important non-infectious inflammatory condition of the lower respiratory tract in horses. Among the hallmarks of EA is hypersecretion^1–5^, often leading to severe mucus plugging of the airways in severe equine asthma (SEA)^4,6–9^. Due to its striking similarities with human asthma, EA is considered a valuable model and has been named accordingly^10,11^.

Two distinct clinical phenotypes of EA are recognized based on their clinical severities^1,12^: mild to moderate equine asthma (MEA), formerly referred to as inflammatory airway disease (IAD), and SEA, previously known as recurrent airway obstruction (RAO)^1^. Both forms differ in their clinical presentation and cytological findings from bronchoalveolar lavage fluid (BALF)^1^. MEA may occur at any age but typically affects young to middle-aged horses. It is characterized by occasional coughing and reduced performance without increased respiratory effort at rest^1^. SEA, on the other hand, predominantly affects horses older than seven years of age and presents with frequent coughing, exercise intolerance, and marked respiratory effort at rest (dyspnea)^1^. BALF cytology is crucial for the clinical diagnosis with only a mild increase in neutrophils, eosinophils, and/or metachromatic cells in MEA but moderate to severe neutrophilic influx in SEA^1^. Excessive mucus accumulation in the tracheobronchial tree is a shared feature of both forms^1^.

Under healthy conditions, mucus forms a protective gel layer that supports hydration, immune defense, and clearance of inhaled particles. It primarily consists of water and solid components, with mucins being essential structural elements^13^. In the human respiratory tract, the gel-forming mucins Mucin 5AC (MUC5AC) and Mucin 5B (MUC5B) constitute the upper (gel) phase of the two-layered airway mucus^13^. They are secreted as large, polymeric glycoproteins in a tissue- and condition-specific manner^13^. Membrane-tethered mucins including Mucin 1 (MUC1), Mucin 4 (MUC4), Mucin 16 (MUC16), and Mucin 20 (MUC20) directly decorate epithelial cell surfaces and form the periciliary brush layer^14^. Non-mucin proteins, such as defensive proteins, growth factors, structural proteins or glycoproteins also contribute to the structure and function of the mucus layer^15^.

The composition and function of mucus are altered in respiratory conditions such as human asthma or cystic fibrosis. Here, mucus constituents including mucins, plasma proteins, inflammatory cells, DNA, actin, and bacteria differ depending on the respective airway disease^13^. Excessive mucus production, along with impaired clearance, leads to airway obstruction and contributes to disease complexity and progression.

Similar dysfunctions have been observed in the airways of horses with asthma. In SEA, both quantitative and qualitative changes in mucus glycoproteins have been reported^16^. Furthermore, unlike healthy horses, those with SEA exhibit increased mucus viscoelasticity following environmental challenge (e.g., stabling with straw bedding and hay feed), along with reduced mucociliary clearance^17^. MUC5B and MUC5AC have been identified as key components of equine airway mucus under diseased conditions^18–20^.

Despite the decisive role of mucus in the pathophysiology of EA, little is known about the proteomic composition of equine airway mucus and its alterations in asthmatic versus healthy horses. Several studies have analyzed the total BALF proteome, yet without specific isolation steps targeting mucus only^21–24^. In this study, we applied label-free quantitative proteomics to isolated BALF mucus pellets to specifically characterize their proteome. We aimed to profile the proteomic compositions of mucus from healthy horses as well as from those affected by MEA or SEA, in order to identify proteins that were differentially abundant between these three groups. Proteins with discriminatory potential between healthy and asthmatics, as well as between the phenotypes, were selected based on Receiver operating characteristic (ROC) curves and areas under the curve (AUC). Two proteins that were increased in both phenotypes and showed high discriminatory potential between healthy and asthmatics were validated in healthy and SEA-affected tissues using immunohistochemistry. In addition to identifying differentially abundant proteins, we used ANOVA followed by median absolute deviation (MAD) analysis to assess non differentially abundant proteins with the lowest variance across all BALF samples, irrespective of health status, as potential references for normalization in quantitative analyses. Overrepresentation analysis using g:Profiler was performed on significantly elevated proteins in diseased horses. Functional enrichment analyses were supplemented by Gene Set Enrichment Analysis (GSEA) of ranked protein lists from the differential abundance analyses to elucidate pathophysiological pathways involved in MEA and SEA. Finally, we correlated the abundance of specific proteins with BALF neutrophil percentages as decisive cytological criterion for the diagnosis of equine asthma.

## Results

### Characteristics of the equine BALF mucus proteome of healthy and asthmatic horses

**Fig. 1:**
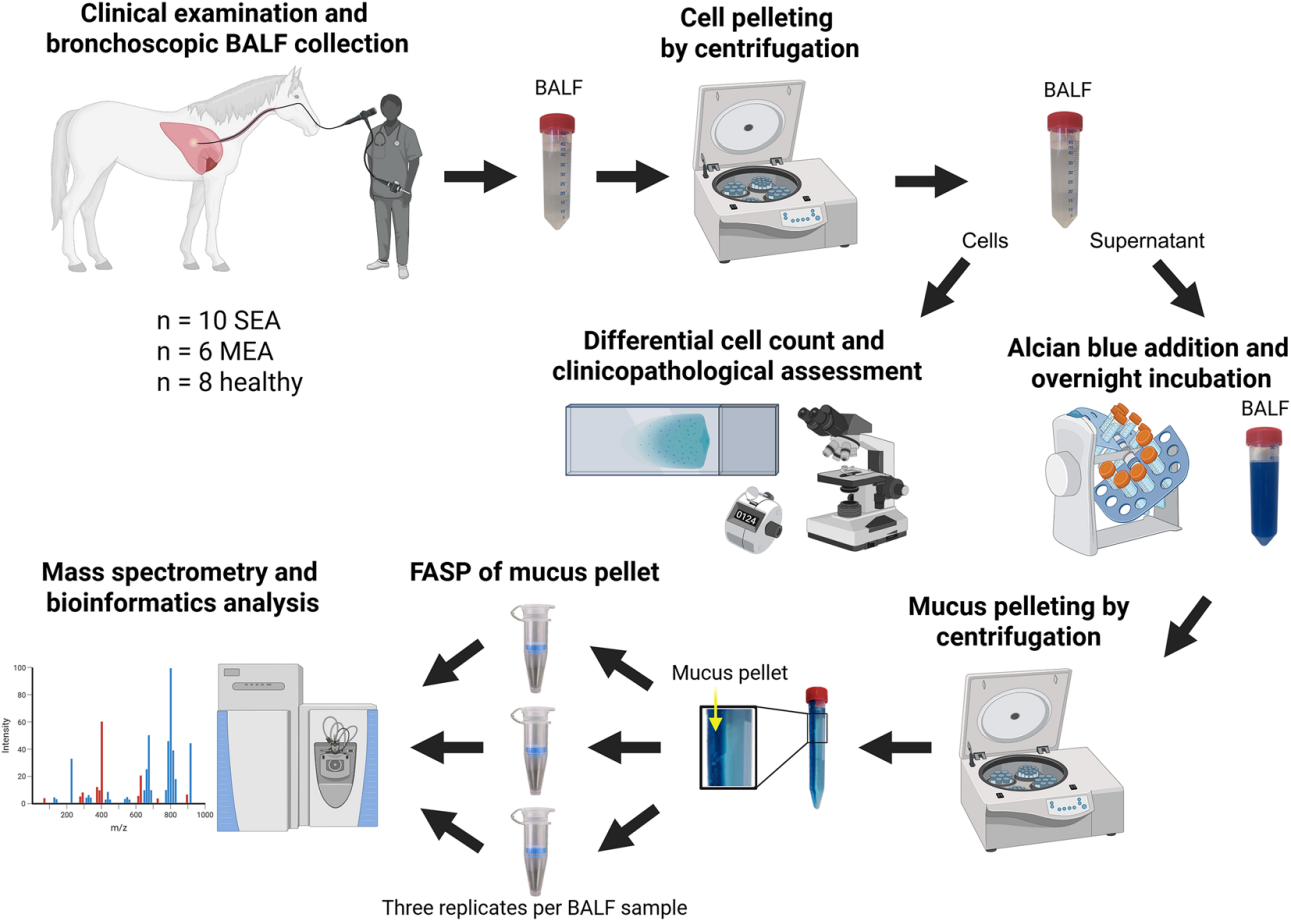
Simplified schematic illustration of the bronchoalveolar lavage fluid (BALF) sample processing and analysis workflow. BALF was collected from horses classified as severe (SEA) or mild to moderate (MEA) equine asthmatic patient or as healthy control, followed by cell pelleting. Supernatants were incubated with Alcian Blue, and mucus was pelleted by centrifugation. Mucus pellets were processed in triplicates by filter-aided sample preparation (FASP) and analyzed by mass spectrometry. Created in BioRender. Freund, A. (2025) https://BioRender.comnprjv2r.

**Fig. 2:**
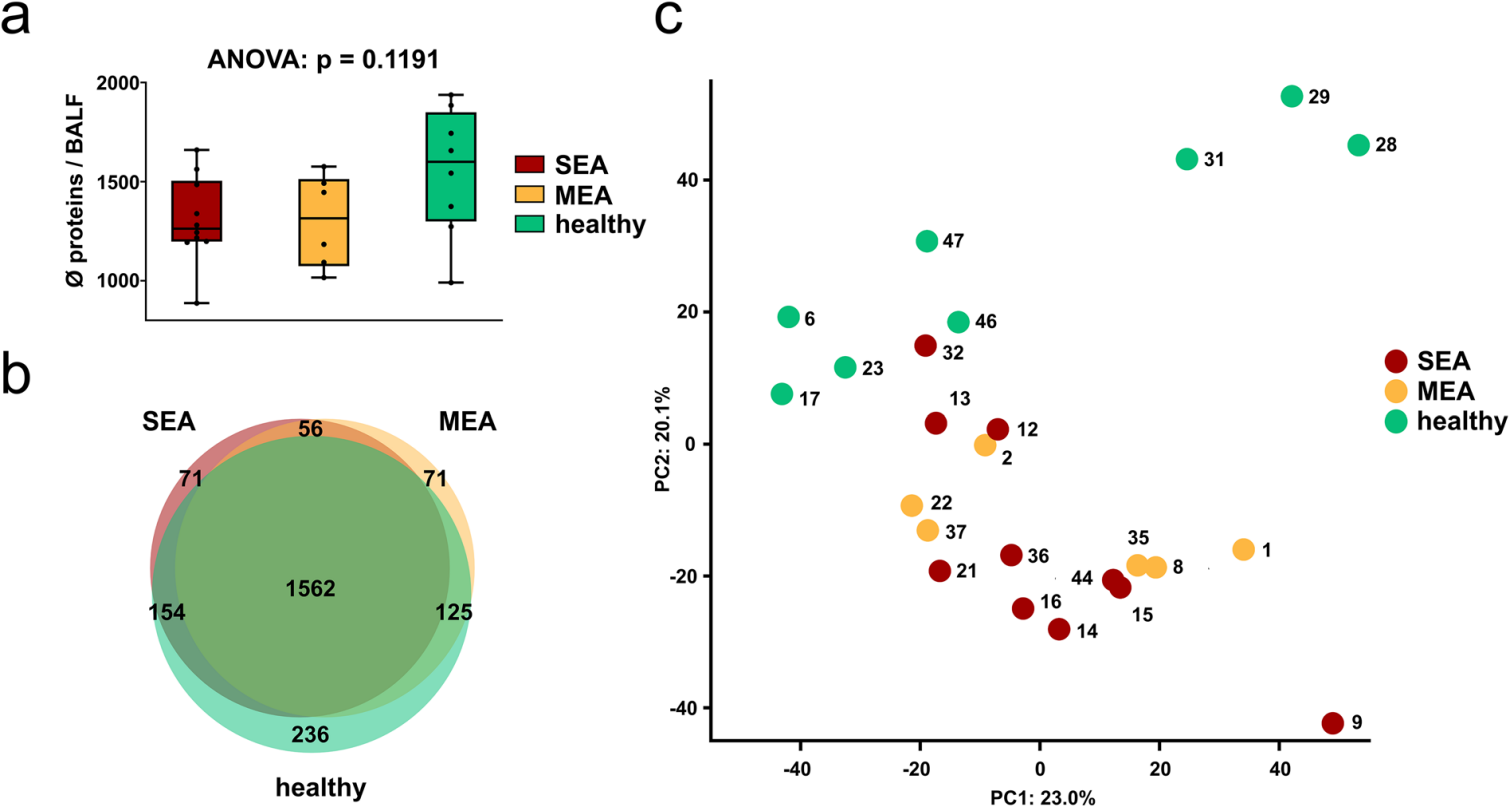
Global and group-specific proteomic characteristics of analyzed BALF mucus samples. (**a**) Number of identified proteins. Each dot represents the counts of proteins per BALF. Boxes represent quartiles and median, and whiskers indicate the range. (**b**) Venn diagram illustrating group-specific and shared proteins among BALF samples from SEA (red), MEA (yellow), and healthy (green) horses. Only proteins were included that were quantified in ≥ 2 technical replicates of at least one BALF per experimental group. (**c**) Principal component analysis (PCA) was performed to assess whether the experimental groups could be distinguished based on their protein intensity profiles.

By applying label-free quantitative proteomics as illustrated in Fig. 1, a total of 2,275 proteins were identified and quantified in BALF mucus samples from healthy and asthmatic horses (Supplementary Table 3). No statistically significant differences in overall counts of identified proteins were observed between SEA, MEA, and healthy groups, however, there was a trend towards a lower number of overall expressed proteins in asthmatic horses (Fig. 2a).

All three groups shared a total of 1,562 quantified proteins. Nevertheless, a selection of certain proteins was only detected in the mucus of SEA (*n* = 71), MEA (*n* = 71) or healthy horses (*n* = 236, Fig. 2b). Principal component analysis separated healthy from asthmatic BALF samples along PC1 (23.0%) and PC2 (20.1%), but did not discriminate between the SEA and MEA subgroups (Fig. 2c). Notably, one BALF sample classified as SEA (BALF 32) clustered closely with the healthy group. Clinical records indicated that this horse met the inclusion criteria for SEA diagnosis but was in remission at the time of sampling. In this context, remission refers to a clinically and/or cytologically improved state compared to a previously documented episode of exacerbation within a recent time frame.

**Fig. 3:**
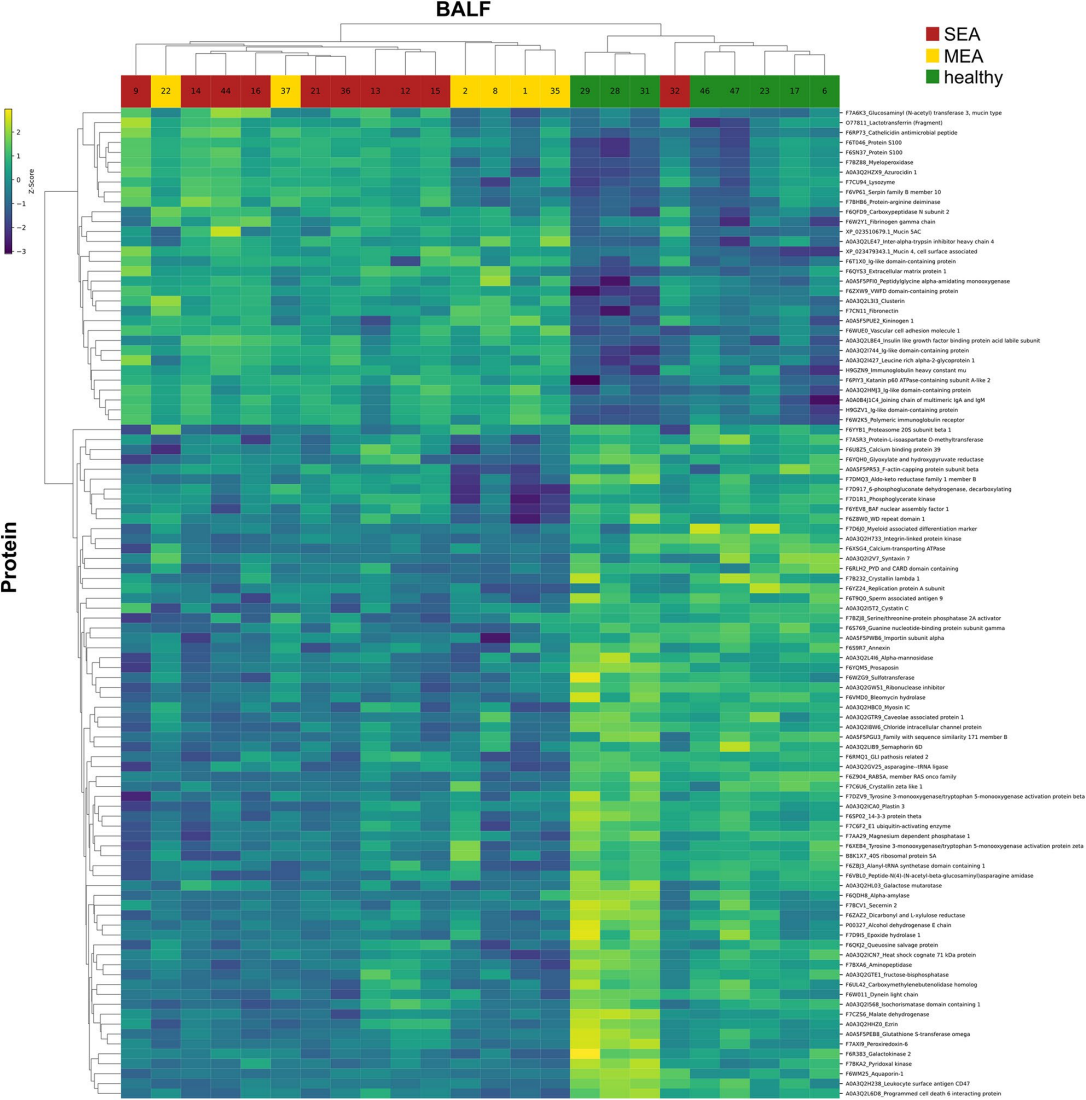
Clustering of ANOVA selected BALF proteins highlights group specific differences in equine asthma. Hierarchical clustering was applied to z-score normalized log₂ LFQ intensity data (Euclidean distance, average linkage). Depicted are the top 100 proteins ranked by their lowest ANOVA p-value. Tukey’s Honest Significant Difference test was used to assess pairwise group differences.

In accordance with the results of the PCA analysis, hierarchical clustering based on the 100 proteins with the lowest adjusted p-values, as identified by ANOVA, revealed distinct segregation of BALF samples into healthy and asthmatic groups, but did not differentiate between the two asthma phenotypes SEA and MEA (Fig. 3). Two main clusters of protein abundances were apparent based on z-score normalized values. In one cluster, approximately one third of these proteins showed a relative increase in BALFs from asthmatic animals, whereas in the second cluster the majority with roughly two thirds of proteins were relatively increased in samples from healthy horses (Fig. 3). Consistent with the PCA findings (Fig. 2c), SEA sample BALF 32 clustered together with the healthy group (Fig. 3).

### Quantitative differences, similarities and discriminatory potential of mucus proteins in healthy and asthmatic horses

**Fig. 4:**
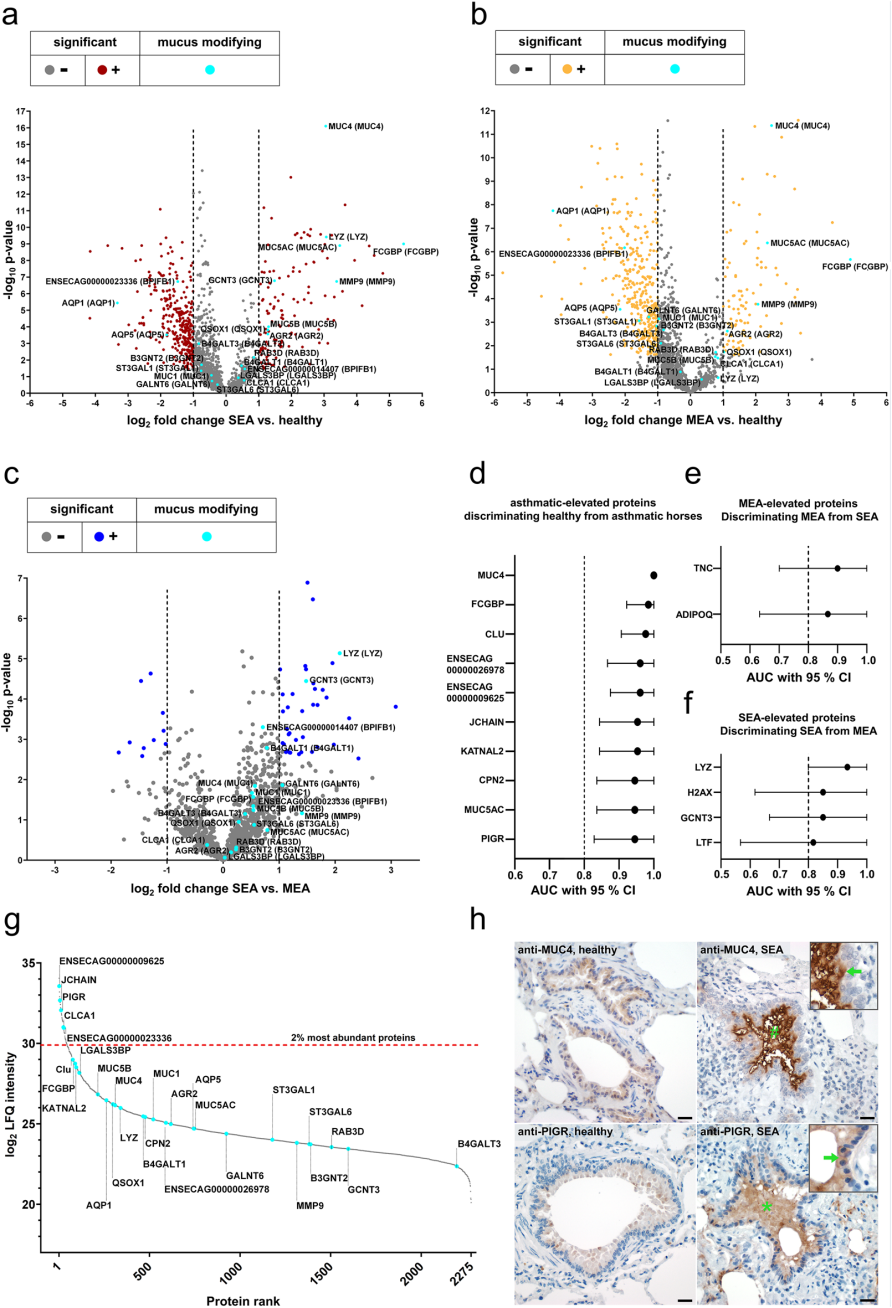
Differentially abundant proteins in SEA and MEA and their discriminatory potential between healthy and asthmatic conditions. (**a-c**) Volcano plots illustrate significantly increased or decreased proteins (log_2_ fold change ≥ 1 or ≤ −1, q-value < 0.05, s0 = 0.1) in red (SEA vs. healthy), yellow (MEA vs. healthy), and dark blue (SEA vs. MEA) dots. Mucus-modifying proteins are highlighted in light blue and labeled with equine gene symbols, with human homologs indicated in parentheses. Alpha-amylase fell outside the displayed x-axis range (**a**, log_2_ fold change = −7.8; -log p-value = 12.38) (**d**) Area under the curve (AUC) values determined by Receiver operating characteristic analysis with 95% CI of the top ten asthma-increased proteins with highest discriminatory potential between healthy and asthmatic (SEA + MEA) horses based on highest AUC values. (**e-f**) AUC values with 95% CI of MEA elevated (**e**) or SEA elevated (**f**) proteins with highest discriminatory potential between SEA and MEA. (**g**) Waterfall plot ranking proteins by their arithmetic mean of log_2_ LFQ intensity values. Mucus-modifying proteins and proteins with discriminatory potential based on Receiver operating characteristics analyses are highlighted. (**h**) Representative photomicrographs of immunohistochemical stained bronchioles from healthy horses (left) and SEA horses (right) using anti-MUC4 (upper panel) or anti-PIGR (lower panel) antibodies. # and * highlight the immunoreactive mucus plugs and arrows point to the apical membrane (anti-MUC4, SEA) or cytosol (anti-PIGR, SEA) of bronchiolar epithelial cells. Color was developed using DAB. Bars represent 20 μm.

To further characterize quantitative differences in the mucus protein composition, pairwise group comparisons (SEA vs. healthy, MEA vs. healthy and SEA vs. MEA) were performed using Student’s t-tests and visualized using volcano plots. Unless stated otherwise, proteins were considered significantly increased or decreased when they exhibited a log₂ fold change ≥ 1 or ≤ − 1, respectively, with a q-value < 0.05 (with s0 = 0.1). A total of 130 proteins in the SEA group and 103 in the MEA group were increased compared to healthy controls (Fig. 4a, b, Supplementary Table 3). In the direct comparison between SEA and MEA (Fig. 4c, Supplementary Table 3), 38 proteins were elevated in SEA, while ten proteins were increased in MEA.

Due to the key role of mucins in the structural and functional properties of airway mucus, particular attention was paid to this protein family. MUC5AC and Mucin 5B (MUC5B) were detected in all groups with higher levels in asthmatic horses compared to healthy controls (Fig. 4a, b, Supplementary Table 3). MUC5AC was significantly elevated in both asthma phenotypes compared to healthy controls (Fig. 4a, b, Supplementary Table 3), while MUC5B was significantly increased in SEA only (Fig. 4b, Supplementary Table 3).

In addition to these gel-forming mucins, the membrane-tethered mucins Mucin 1 (MUC1) and MUC4 were detected across all groups (Fig. 4a, b, Supplementary Table 3). Asthmatics showed a reduction in MUC1 only in the MEA group, but with a log_2_ fold change slightly below 1 (log_2_ fold change − 0.96, Fig. 4a, b, Supplementary Table 3) compared to healthy controls. No significant difference of MUC1 abundance was observed between SEA and MEA. In contrast, MUC4 was significantly and strongly increased in both asthma phenotypes compared to healthy controls (Fig. 4a, b, Supplementary Table 3), suggesting a previously unrecognized role in EA. No significant difference in MUC4 abundance was observed between SEA and MEA (Supplementary Table 3), indicating that this increase may be a general feature of equine asthma.

Beyond these mucins, several additional proteins associated with structural integrity of mucus, mucus production or modification (here referred to as mucus-modifying proteins) exhibited group-specific differences in their relative abundance. Among these, FCGBP, Matrix Metalloproteinase 9 (MMP9), Anterior Gradient 2, Protein Disulphide Isomerase Family Member (AGR2), and Quiescin Sulfhydryl Oxidase 1 (QSOX1) were significantly elevated in both MEA and SEA (Fig. 4a, b, Supplementary Table 3) compared to healthy controls. In a direct comparison between SEA and MEA, only LYZ and GCNT3 were found to be significantly more abundant in SEA (Fig. 4c, Supplementary Table 3). Interestingly, Aquaporin 1 (AQP1), Aquaporin 5 (AQP5), and ENSECAG00000023336 (human homolog: BPIFB1) were significantly decreased in both phenotypes compared to healthy controls (Fig. 4a, b, Supplementary Table 3). Acetylglucosaminyltransferase 2 (B3GNT2), Polypeptide N-Acetylgalactosaminyltransferase 6 (GALNT6), and ST3 Beta-Galactoside Alpha-2,3 Sialyltransferase 1 (ST3GAL1) were reduced only in MEA compared to healthy controls (Fig. 4a, Supplementary Table 3).

To assess the ability of proteins with higher abundance in asthmatic horses to discriminate between diseased and healthy animals, ROC analysis was performed and the area under the curve was calculated. Following established criteria for diagnostic test performance, proteins with an AUC between 0.8 and 0.9 were considered to have good discriminatory potential, whereas AUC values > 0.9 indicated excellent discrimination^25^. In total, 80 proteins that were increased in asthmatic compared to healthy horses showed an AUC above 0.8, including 24 with excellent discriminatory potential (Supplementary Table 4). The latter included Mucin 4 (MUC4), Fc Gamma Binding Protein (FCGBP), Clusterin (CLU), Joining Chain of Multimeric IgA and IgM (JCHAIN), Katanin P60 ATPase-Containing Subunit A-Like 2 (KATNAL2), Carboxypeptidase N Subunit 2 (CPN2), Mucin 5AC (MUC5AC), Polymeric Immunoglobulin Receptor (PIGR), and two immunoglobulin fragments (ENSECAG00000009625, ENSECAG00000026978, Fig. 4d). Furthermore, in MEA patients, Tenascin C (TNC) and Adiponectin (ADIPOQ) were elevated and demonstrated potential to differentiate them from healthy and SEA patients (Fig. 4e). Conversely, Lysozyme (LYZ), Histone H2A (H2AX), Glucosaminyl (N-Acetyl) Transferase 3, Mucin Type (GCNT3) and Lactotransferrin (LTF) were elevated in SEA patients, with discriminatory potential from MEA and healthy horses (Fig. 4f).

A waterfall plot, ranking proteins by their mean log_2_ LFQ intensity values across all BALF samples, was generated to provide a comparative overview of the relative expression levels of all quantified proteins and all mucus-modifying proteins and the top ten proteins with the highest discriminatory potential between asthmatic and healthy horses were highlighted (Fig. 4g). The plot illustrates that these proteins were consistently detected with sufficient intensities, supporting their robustness for subsequent analyses (Fig. 4g, Supplementary Table 3). The immunoglobulin component ENSECAG00000009625, JCHAIN, PIGR, Calcium-Activated Chloride Channel Regulator 1 (CLCA1) and ENSECAG00000023336 (human homolog: BPIFB1) were among the 2% most abundant proteins (Fig. 4g, Supplementary Table 3).

To further validate the LC-MS findings, two proteins increased in SEA for which cross-reactive antibodies were commercially available, MUC4 and PIGR, were localized by immunohistochemistry in healthy and diseased tissues. Both proteins were significantly elevated in diseased horses according to the proteomic data and showed excellent discriminatory potential (Fig. 4d). Immunohistochemistry was performed on tissues from healthy controls and SEA horses, reflecting the availability of well-preserved samples only for the latter phenotype. In line with the LC-MS findings, both proteins were strongly detected in asthmatic horses. MUC4 was predominantly localized to the apical membranes of bronchial epithelial cells and within mucus plugs, while PIGR was detected in the cytosol of bronchial epithelial cells as well as in mucus plugs of asthmatic horses (Fig. 4h).

In addition to the analysis of differentially abundant proteins, we further asked which non differentially abundant proteins exhibited the least variance across all BALF samples, irrespective of health status or disease phenotype. With this approach, we aimed to identify proteins that were consistently detected at similar levels, which would make them useful candidates as references for normalization in quantitative assays. To this end, we calculated the median absolute deviation of the log₂ transformed LFQ intensities for each protein across all BALF samples. Additionally, only proteins without significant differences between the SEA, MEA, and healthy groups according to ANOVA (*p* > 0.05) were reported. Only the top ten proteins with the lowest MAD values are reported here (Table 1). Pyruvate kinase, Actin related protein 3 or Eukaryotic translation elongation factor 2 showed minimal inter-sample variability, indicating a uniform presence of these proteins in BALF mucus.

**Table 1:** Top ten proteins with the lowest variability of log_2_ LFQ intensity values (as measured by median absolute deviation, MAD).

Accession	Protein name	Median log_2_ LFQ intensity value	MAD
B3IVM1	Pyruvate kinase	28.23	0.13
F7DE06	Annexin	25.96	0.14
F7B9Q9	Protein kinase C and casein kinase substrate in neurons 2	23.93	0.15
F6WE31	Actin-related protein 2/3 complex subunit 3	26.46	0.16
A0A5F5PLR4	2,3-cyclic-nucleotide 3-phosphodiesterase	24.54	0.17
F6W354	Actin related protein 3	26.92	0.17
F7DTB6	Karyopherin subunit beta 1	25.12	0.17
A0A5F5PT22	Integrin beta	25.97	0.17
A0A3Q2IB08	Eukaryotic translation elongation factor 2	26.69	0.17
F6WWW7	F-actin-capping protein subunit alpha	26.21	0.19

### Functional enrichment analyses

**Fig. 5:**
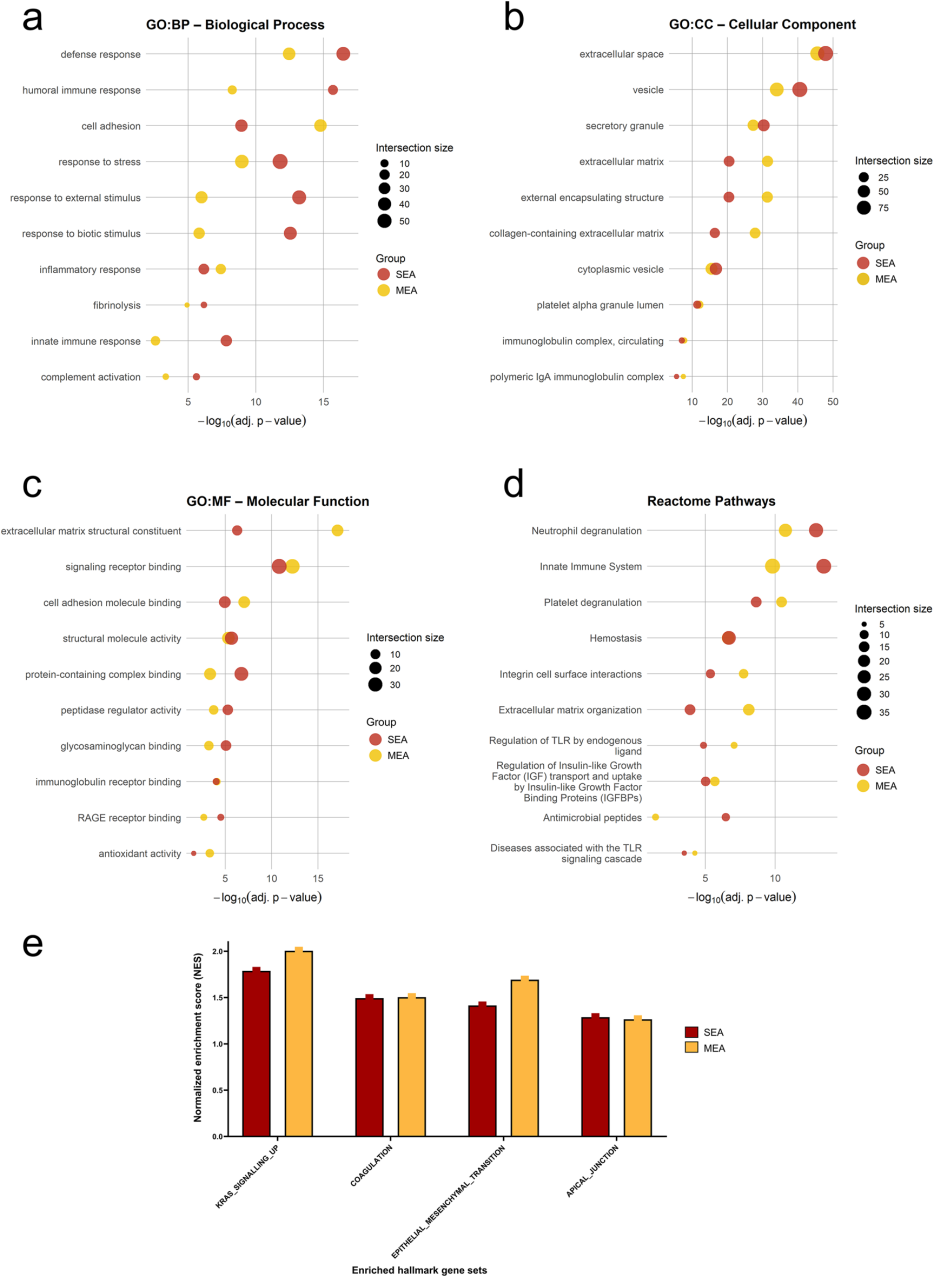
Functional enrichment analysis of proteins detected in BALF mucus of SEA and MEA horses. Significantly increased proteins (log_2_ fold change ≥ 1 or ≤ −1, q-value < 0.05, s0 = 0.1) from SEA and MEA groups were subjected to overrepresentation analysis using Gene Ontology (GO, **a-c**) and Reactome pathway annotations (**d**). Displayed are the top ten nonredundant terms or pathways. (**a**) GO: Biological Process (BP) terms enriched in SEA (red) and MEA (yellow) samples. (**b**) GO: Cellular Component (CC) terms (**c**) GO: Molecular Function (MF) terms illustrating functional protein properties. (**d**) Reactome pathway enrichment. The x-axis indicates - log₁₀ adjusted p-values, the section size is reflected by the sizes of the dots. (**e**) Gene Set Enrichment Analysis (GSEA) on ranked protein lists from the differential abundance analyses of SEA and MEA relative to healthy controls. Normalized enrichment scores (NES) of selected positively enriched Hallmark gene sets (MSigB v2024.1, h.all.v2024.1.Hs.symbols.gmt) in SEA (red) and MEA (yellow) compared to healthy controls. Displayed are the four significantly enriched pathways across both groups.

To characterize the biological context of the proteins significantly enriched in MEA and SEA, GO-term and Reactome pathway overrepresentation analyses were employed, where significance reflects the adjusted p-value and intersection size denotes the number of input proteins assigned to the respective term. In the category of Biological Processes (GO:BP), enriched terms in both SEA and MEA included “defense response”, “response to stress”, and “response to external stimulus” (Fig. 5a). SEA was more strongly associated with the majority of enriched terms. Only the terms “cell adhesion” and “inflammatory response” were more strongly enriched in MEA (Fig. 5a). These data point towards an inflammation-associated proteomic profile in either phenotype.

In the Cellular Component category (GO:CC), adjusted p-values were similarly distributed between the phenotypes with both showing strong enrichment for “extracellular space”, “vesicle”, and “secretory granule” (Fig. 5b). MEA showed higher enrichment in “extracellular matrix”, “external encapsulating structure”, and “collagen-containing extracellular matrix” compared to SEA, suggesting a more pronounced activation of airway remodeling in MEA.

Similarly, Molecular Function terms (GO:MF) such as “signaling receptor binding”, “cell adhesion molecule binding”, “structural molecule activity” and “peptidase regulator activity” were enriched in both groups (Fig. 5c). However, “extracellular matrix structural constituent” was particularly enriched in MEA horses, further reflecting enhanced matrix-related remodeling.

Reactome pathways revealed strong enrichment of “Neutrophil degranulation” and “Innate Immune System” pathways in both groups (Fig. 5d), aligning with the known cytopathological characteristics of EA and the cytopathological characteristics of the cohort investigated in this study. These two pathways were slightly more strongly enriched in SEA. In contrast, MEA showed stronger associations with “Platelet degranulation”, “Extracellular matrix organization”, and “Formation of fibrin clot” (Fig. 5d), possibly suggesting alterations in hemostatic regulation or immune complex formation and immunoglobulin effector function. A complete list of overrepresented GO terms and Reactome pathways is provided in Supplementary Table 5.

In addition to the GO term and Reactome pathway analysis, a gene set enrichment analysis (GSEA) was performed on ranked protein lists from the differential abundance analyses of SEA and MEA relative to healthy controls. In contrast to the GO-term and Reactome analyses, GSEA considered not only strongly upregulated proteins with a fold change ≥ 2 in SEA vs. healthy or MEA vs. healthy comparisons. It also included proteins that were decreased in SEA vs. healthy or MEA vs. healthy comparisons and applied no threshold for the fold change. This approach enables the detection of pathway-level shifts driven by moderate changes. Among the positively enriched gene sets, both SEA and MEA samples showed consistent activation of inflammation- and stress-associated pathways.

Notably, the pathways KRAS_SIGNALING_UP, COAGULATION, EPITHELIAL_MESENCHYMAL_TRANSITION (EMT), and APICAL_JUNCTION were significantly enriched in both groups (Fig. 5e). The highest normalized enrichment score (NES) was observed for KRAS_SIGNALING_UP (NES = 2.01, FDR = 0.001), followed by EMT (NES = 1.69, FDR = 0.008), both in MEA. In SEA, KRAS signaling was also highly enriched (NES = 1.79, FDR = 0.006), accompanied by exclusive significant enrichment of the COMPLEMENT (NES = 1.31, FDR = 0.248) pathway (Supplementary Table 6), which may reflect humoral immune activation in the more severe phenotype. Full GSEA reports for both groups are provided in Supplementary Table 6. The enrichment of KRAS_SIGNALING_UP, EPITHELIAL_MESENCHYMAL_TRANSITION (EMT), and APICAL_JUNCTION pathways reflect the findings from the overrepresentation analysis and accordingly point towards enhanced airway remodeling and regulation of epithelial barrier dysfunction in equine asthma.

### Correlation of proteomic data with BALF neutrophil counts

**Fig. 6:**
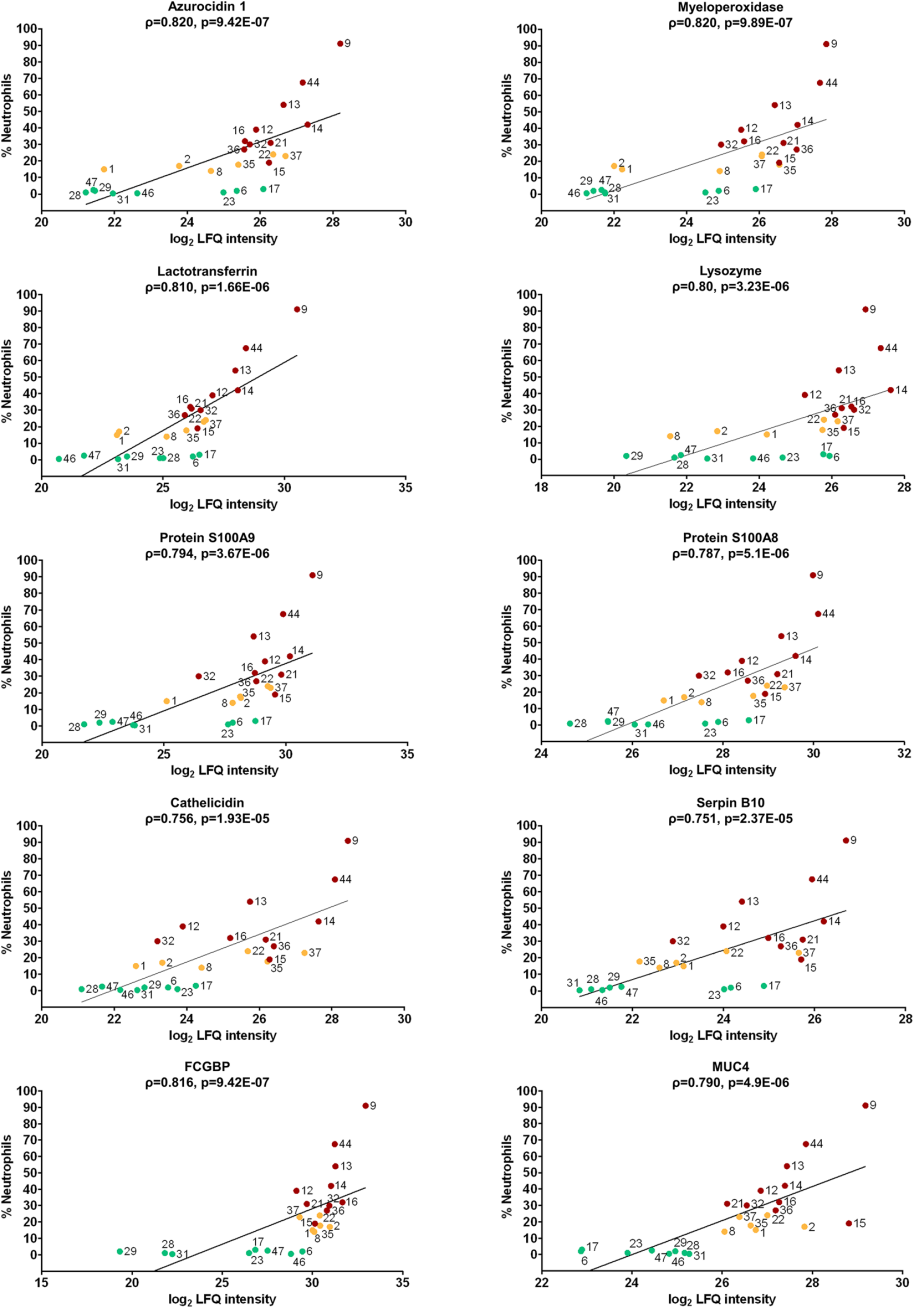
Correlation of protein abundance with BALF neutrophil counts. Scatter plots showing the correlation between neutrophil percentage and log₂ transformed LFQ intensities for the ten proteins with the highest absolute positive Spearman correlation coefficients. The proteins are arranged by protein group and decreasing correlations (ρ), with eight neutrophil-associated (upper panels) and two mucus-modifying proteins (MUC4, FCGBP, lower panel). Each dot represents the mean log_2_ LFQ intensity obtained from three technical replicates per BALF sample. Green = healthy, red = SEA yellow = MEA. The number next to each dot indicates the corresponding BALF sample ID (Supplementary Table 1). Spearman ρ and p-values are indicated in each plot.

Cytology is a central criterion in the diagnosis of equine asthma. Specifically, a moderate to marked increase in neutrophils in BALF of asthmatic horses is a key feature of this disease and helps to discriminate between the two EA-phenotypes. Thus, we aimed to identify proteins correlating with the proportion of neutrophils in the cytology samples of the same horses. For each protein that was identified and quantified in at least 50% of BALF samples within at least one experimental group (SEA, MEA or healthy), we calculated the Spearman’s rank correlation coefficient (ρ) between log_2_ LFQ intensity and the percentage of neutrophils. This non-parametric measure of correlation ranges from − 1 (perfect negative association) to + 1 (perfect positive association). According to Cohen’s classification, correlations with ρ > 0.5 are considered strong^26^. The ten proteins with the highest positive correlation coefficients were selected. All showed a significant and strong positive correlation with neutrophil percentages (ρ = 0.751–0.82, *p* < 0.001, Fig. 6). These proteins included canonical neutrophil-associated, but not in all cases neutrophil-exclusive proteins, such as Azurocidin 1, Myeloperoxidase, Lactotransferrin, Lysozyme, Protein S100A9, Protein S100A8, Cathelicidin and Serpin B10. Interestingly, MUC4, a mucin protein, and FCGBP, a mucus-associated glycoprotein, also exhibited strong positive correlations (Fig. 6, Supplementary Table 7). Myeloperoxidase, Cathelicidin, FCGBP, S100A9 and S100A8 were significantly and strongly negatively correlated with macrophage percentages with ρ < −0.5 (Supplementary Table 7). No strong correlations of the ten proteins depicted in Fig. 6 with lymphocyte percentages were detected (Supplementary Table 7).

Similar to the ROC analyses, it became obvious that MUC4 allowed for the most distinct separation between healthy controls and asthmatic horses when visualized by plotting the ratios of neutrophil counts to log_2_ LFQ protein intensities (Fig. 6). This finding alludes to a possible role as a sensitive marker for disease related alterations in the airway mucus proteome.

## Discussion

Mucus hypersecretion is a characteristic finding in EA and is typically seen in both of the two severity-based clinical phenotypes, MEA and SEA^1–5^. Moreover, severe mucus accumulation results in mucus plugs in small airways which is an established histomorphological diagnostic criterion in SEA^4,6,7,9,27^. Airway mucus as direct interface between environmental determinants and the airway epithelium represents a decisive barrier structure where disease associated processes are initiated, modulated and maintained on the molecular level. We therefore hypothesized that improved knowledge of compositional changes in EA-associated heterostasis of airway mucus may lead to a better understanding of underlying mechanisms, and ultimately to more specific diagnostic and therapeutic actions.

Several authors have analyzed the overall equine BALF proteomes in the context of EA, but did not analyze isolated mucus alone^21,23,24^. Instead, these investigations mainly focused on linkage analyses to investigate pathways associated with interleukin 4 receptor, molecular events associated with neutrophilic airway inflammation or on comparative functional modelling based on GO terms^21,22,24^. Another proteomic study examined BALF as a whole, without separation of mucus, and identified approximately 30 differentially expressed proteins and found proteins such as secretoglobin and transferrin^23^. Of note, no mucins were identified in any of these studies.

Functional enrichment analysis revealed a proinflammatory and metaplastic proteomic signature across both asthma phenotypes, aligning well with the clinical presentation and current understanding of equine asthma pathophysiology. Further, the enrichment of GO terms and Reactome pathways related to extracellular matrix (ECM) components implicated tissue remodeling in the lower airways of horses affected by EA. Unlike in some forms of human asthma where basement membrane thickening is well established^28,29^, such changes have not yet been reported in EA. Instead, SEA has been associated with an increase of lamina propria ECM^30,31^, including alterations in collagen composition and enhanced deposition of elastic fibers^30^. Additionally, airway smooth muscle cells are hyperplastic and hypertrophic in SEA patients^32,33^, accompanied by goblet cell hyperplasia or metaplasia^34,35^. Recent histomorphometric investigations have identified similar changes in MEA patients, including thickening of the lamina propria, smooth muscle fibrosis, and epithelial hyperplasia^36^. To date, however, the effect sizes of these changes have not been directly compared between the two EA phenotypes which limits a thorough validation of the pathway findings within the scope of this study.

Further, we identified several EA-associated changes in isolated mucus on the proteomic level that reveal a detailed spectrum of compositional alterations with probable connections to rheological and immunological heterostasis.

Mucin proteins are key structural components of mucus. In this study, the gel-forming airway mucins MUC5AC and MUC5B were detected in BALF mucus pellets of healthy and diseased horses. MUC5B was significantly increased only in SEA compared to healthy horses, while MUC5AC was found significantly increased in horses with either form of the disease. The latter finding is consistent with previous studies in human asthma and animal models of asthma, where upregulation or higher abundance of the gel-forming MUC5AC has been observed^29,37–40^. Likewise, *MUC5AC* mRNA has been found upregulated in horses with asthma^20^. In both human asthma and animal models of asthma, the abundance of MUC5B, however, appeared to be variable^29,40,41^.

Beyond the identification of the membrane-tethered mucins MUC1 and MUC4, we also detected transmembrane and intracellular proteins in the mucus pellet proteome. While such findings could in part result from epithelial turnover or immune cells adhering to the highly viscous mucus, alternative explanations must also be considered. For example, membrane proteins can enter the extracellular space via proteolytic cleavage. This mechanism has been described for MUC1 and MUC4. Both mucins possess extended extracellular domains that form a protective steric barrier at the epithelial surface^14^. These domains are known to be released from the epithelial surface by proteolytic cleavage^42–46^. Other membrane-associated proteins may enter the mucus compartment via extracellular vesicles or membrane shedding, supporting this, an in-vitro study using carefully collected mucus from human tracheal tissue maintained under cell culture conditions reported that the mucus contained predominantly transmembrane and cytosolic proteins^47^. These findings suggest that such components may represent bona fide elements of the mucus proteome.

Notably, MUC4 was significantly and strongly elevated in both EA phenotypes, SEA and MEA, with excellent discriminatory potential between healthy and diseased animals. However, the role of MUC4 in asthma is still unclear. In humans, MUC4 has been associated with epithelial regeneration and differentiation in asthmatic airways^48,49^. Furthermore, and in line with our findings, preliminary evidence has suggested overexpression of MUC4 by bronchial epithelial cells in severe human asthma^50^. In comparison, MUC1 levels were not increased in asthmatic horses, and no association between MUC1 expression and distinct inflammatory phenotypes of asthma has been described in humans. In a recent study using bulk RNA-seq from BALF cell pellets collected from horses with SEA, MEA, and healthy controls, *MUC1*, *MUC4*, and *MUC5AC* transcripts were found downregulated in SEA^51^. We interpret this difference to our immunohistochemical findings with strong MUC4 specific signals on the apical membrane of bronchiolar epithelial cells as well as within the mucus plugs in SEA affected horses as reflecting complementary readouts rather than a contradiction. Based on their findings, the authors have proposed that mucus plug formation in SEA may result primarily from impaired mucociliary clearance rather than increased mucus secretion^51^. In this context, it appears noteworthy that mucins are produced by epithelial cells. However, the sample matrix used in that study consisted of RNA extracts from leukocyte dominated BALF cell pellets with only very few epithelial cells (0–3.9%)^51^. Therefore, this discrepancy seems to be based on cell-composition confounding effects in that the bulk RNA-Seq data derived from those extracts likely contained few epithelial transcripts but overwhelmingly numerous leukocyte transcripts. In human asthmatic patients, *MUC5AC* mRNA was increased in bronchial biopsy specimens and epithelial brushings, supported by elevated MUC5AC protein in induced sputum^29,40,41,52^. Similarly, and consistent with our findings, *MUC5AC* mRNA was increased in homogenized airway tissue from SEA affected horses^20^. Together, these observations suggest that BALF pellet transcriptomics and immunohistochemical investigations of bronchial tissue capture different facets of respiratory mucin pathophysiology. Furthermore, neither pellet transcriptomics nor LC-MS and IHC measure mucociliary clearance which would require functional assessments. Future work should clarify whether mucus plugging is driven predominantly by reduced clearance, increased mucin production, or both.

In addition, mucus-associated proteins such as FCGBP and AGR2 which closely interact with intestinal MUC2^53–57^ were strongly increased in SEA and MEA mucus samples. Consistently, the protein disulfide isomerase AGR2 has been found induced in human asthma and it is thought to contribute to overproduction of the airway mucins MUC5AC and MUC5B^57^.

Mucins carry dense O-glycans attached to serine/threonine (Ser/Thr) residues^58^. Mucin-type O-glycosylation is a complex biosynthetic pathway involving various enzymes^59^. It is initiated by N-Acetylgalactosamine (GalNAc)-transferases such as GALNT6 that transfer GalNAc to Ser/Thr to generate the Tn-antigen^59^. Subsequent glycosyltransferase reactions to this Tn-antigen elaborate four different core structures^59^. For example, the mucin-type core 2 branch is formed by GCNT3^59^. These core O-glycans may then be extended by the addition of polylactosamine (poly-LacNAc) by B3GNT2^60^ or terminally modified by α2,3-sialylation by ST3GAL1^61^. Since O-glycosylation is critical for hydration, charge, and viscoelasticity of mucus^57^, alterations in glycosylation profiles may directly impact mucus hydration and viscosity^62–65^. As part of our results, the glycosyltransferase GCNT3, was severely increased in SEA supporting core 2-rich, complex glycosylation of mucins. In contrast to GCNT3, several glycosyltransferases were significantly reduced only in MEA compared to healthy horses, such as GALNT6, ST3GAL1 and B3GNT2 which may promote a less organized mucus glycosylation. However, alterations in glycosylation are just one factor affecting mucus viscosity, and protein abundance does not necessarily reflect enzymatic activity.

Interestingly, it has been proposed that the mucus-modifying protein LYZ increased the viscosity of airway mucus^66^. The high abundance of LYZ in SEA horses may further support the formation of stickier mucus plugs in the airways of these horses. Notably, aquaporins AQP1 and AQP5 were markedly less abundant in the mucus of both asthma phenotypes compared to healthy controls. The decrease of these aquaporins has also been reported in rat models of asthma^67^. These proteins serve as key mediators of endothelial and epithelial water transport^68^ which appear essential for mucus hydration^67^. The role of the reduced abundance of these aquaporins in the context of mucus dehydration as common pathological feature in both SEA and MEA should be investigated further.

Among the mucus-modifying proteins, the Calcium-Activated Chloride Channel Regulator 1 (CLCA1) has also been described in several mammalian species^69–73^. In mice, CLCA1 was even identified as the most abundant airway mucus protein^70^. In horses with SEA, it had been found overexpressed due to goblet cell hyper- and metaplasia^73^. In the present study, the equine CLCA1 was among the 2% most abundant proteins and slightly elevated in both asthmatic groups compared to healthy control, however, not to a statistically significant extent. The polymeric immunoglobulin receptor (PIGR) has recently been reported as a highly abundant protein in the BALF of healthy horses and to be increased in severe asthma^22^. In our study, PIGR was also among the 2% most abundant proteins in BALF mucus and its differential abundance showed discriminatory potential between healthy and asthmatic horses. We furthermore recorded significantly increased levels of PIGR in mucus of both SEA and MEA horses compared to healthy controls. Immunohistochemical staining confirmed this difference and localized PIGR to the luminal mucus plugs and respiratory epithelial cells. In addition to PIGR, the joining chain of polymeric immunoglobulins (JCHAIN) was also markedly increased in asthmatic horses with high discriminatory potential between healthy and asthmatic horses. Both proteins are necessary for the transport of secretory immunoglobulins IgA and IgM across epithelial barriers^74–77^, suggesting enhanced activation of the humoral immune response in equine asthma. This is particularly relevant in neutrophilic phenotypes of EA, where increased levels of Aspergillus-specific IgA have been previously described^78^. Our proteomic data provide molecular evidence for this concept and may indicate a potentially antigen driven humoral immune response as part of airway inflammation, ultimately leading to mucus plugging. Furthermore, numerous immunoglobulin fragments, some highly increased, were identified in the mucus proteome. However, due to the still incomplete annotation of the equine genome, precise assignment to specific immunoglobulin isotypes or subclasses remains challenging.

A clear separation was not apparent between SEA and MEA, neither in the heatmap nor in PCA analyses. The latter represents a global clustering approach primarily driven by major sources of variance and therefore may fail to reveal more nuanced phenotype differences. However, differential abundance and enrichment analyses indicated subtle proteomic differences between the clinical phenotypes. Horses with SEA showed preferential increases of MUC5B, GCNT3, and LYZ, alongside stronger complement and innate pathway signatures as well as neutrophil-associated proteins. These findings are in line with clinical and histopathological changes in SEA patients, where accumulation of hyperconcentrated, sticky mucus has been firmly established along with formation of small airway plugs, often admixed with neutrophils^4,6–9^. By contrast, our MEA results showed slightly stronger enrichment of extracellular matrix organization, platelet degranulation, and a fibrin related pathway together with selective reduction of enzymes relevant to O-glycosylation such as GALNT6, ST3GAL1, B3GNT2. We interpret this pattern as compatible with shorter, less sialylated mucin glycans leading to MEA-specific mucus properties different from altered mucus in SEA and early airway remodeling, as previously reported in MEA affected horses^36^. Thus, while global clustering approaches fail to capture these nuanced signals, pathway level analyses and changes in differential abundance of single proteins point towards mechanisms of established biologically meaningful differences between SEA and MEA. Clearly, these changes in particular warrant detailed investigation in future studies.

In summary, this study provides a detailed proteomic landscaping of isolated airway mucus in healthy and asthmatic horses, revealing phenotype specific alterations in mucin composition, mucus-modifying enzymes, immune effectors and circulation-related components. These findings offer molecular insights into mucus-related pathologies in EA and may support novel diagnostic or even therapeutic strategies. Future work should embark on more details of the subtle phenotype-specific differences between MEA and SEA and include larger cohorts also comprising other, non-asthmatic respiratory conditions with mucus heterostasis to broaden the comparative perspective of airway dysfunction.

## Methods

### Inclusion criteria and sample collection

BALF samples were obtained from healthy (*n* = 8), MEA (*n* = 6) and SEA (*n* = 10)-affected horses. The diagnosis was based on clinical signs, tracheobronchoscopic findings, BALF cytology and arterial blood gas values (Supplementary Table 1). Samples of healthy and diseased horses were collected with permission from local authorities (State Office of Health and Social Affairs Berlin, Germany StN 020/21, L 0269/18 and Landesdirektion Sachsen, Germany, TVV22/20 file number 25–5131/490/23). Asthmatic horses were sampled during routine diagnostic workflows only. All methods were performed in accordance with the relevant guidelines and regulations. The study was conducted in accordance with ARRIVE guidelines (https://arriveguidelines.org).

After clinical examination, horses were sedated with detomidine hydrochloride 0.01 mg/kg IV in combination with either levomethadone hydrochloride 0.05 mg/kg IV or butorphanol tartrate 0.02 mg/kg IV, or with detomidine and butorphanol for endoscopy and collection of BALF. To reduce hyper-reactivity and suppress the coughing reflex, the airways were desensitized by transendoscopic instillation of a local anesthetic (lidocaine hydrochloride 2%, 20–30 ml/horse) in the region of the tracheal bifurcation before BALF collection was performed. Following bronchoscopy grading, descriptive criteria were applied according to established guidelines^27,79–87^. After removal of the endoscope, a flexible BAL catheter with balloon (3 m, 10 mm diameter) was advanced in the lower airways and cuffed by air inflation upon reaching elastic resistance of the bronchioles. 60 ml per 100 kg body mass of prewarmed (37 °C), sterile phosphate-buffered saline (PBS Lonza) was instilled and re-aspirated for further examination. Sample material was considered adequate if ≥ 40% of instilled volume was recovered.

BALF was evaluated macroscopically and the recovered volume was documented. Preparation for cytological examination included centrifugation of the samples (500 x *g* for 10 min). Either smears or cytospins were prepared, air dried and stained with the Diff-Quick method alone or with Diff-Quick and Toluidine blue. Cytology was performed under a light microscope (1,000 x magnification) by clinicians experienced in the diagnostics of equine respiratory disease, under supervision of board-certified specialists (ECEIM) with differential counts based on 400 or 500 cells, respectively. Evaluation included the relative percentages of cells, cell morphology and the presence of foreign material (e.g. pollen, hyphae or crystals).

Diagnosis was based on the revised 2016 ACVIM-Consensus Statement for Equine Asthma^1^. In accordance with the guidelines, healthy horses were defined as having ≤ 5% neutrophils, ≤ 1% eosinophils and ≤ 2% metachromatic cells, with BALF cytology typically dominated by alveolar macrophages. BALF neutrophilia is characteristic of EA. For a cytological diagnosis of SEA, a cut-off value of > 25% was applied^1,88,89^. A diagnosis of MEA was made for samples containing relative percentages of > 10% for neutrophils, > 5% for mast cells and > 5% for eosinophils^90^.

The final diagnosis for each horse was made by taking the clinical examination, blood-gas analysis, respiratory endoscopy and BAL cytology into account by clinicians experienced in the diagnostics of equine respiratory disease and board-certified specialists (ECEIM). In this context, one horse (BALF 15, Supplementary Table 1) had a 19% BALF neutrophil proportion but was nevertheless diagnosed with SEA.

### Mucus pellet isolation and filter aided sample Preparation (FASP)

The experimental workflow is illustrated in Fig. 1. Mucus pellets from horses of the different groups were isolated and processed as described^91^ based on a modified protocol previously described for mice^70^. The person conducting the experiment was blinded. In brief, BALF-cells were removed by centrifugation at 300 x *g* for 30 min. 5% v/v of a saturated Alcian Blue 8GX solution was added to each cell-free BALF sample, incubated overnight at room temperature on a rotator and centrifuged at 14,000 x *g* for 30 min. Mucus pellets were stored at −80 °C until resolubilization as described^70^ followed by passage through a 27 G cannula and incubation in an ultrasonic bath for 20 min.

The resolubilized samples were dialyzed against 0.1 M Tris-HCl, pH 8.5, using Slide-A-Lyzer™ MINI 10 K MWCO units (Thermo Fisher Scientific). Protein concentrations were determined fluorometrically (Qubit™ Protein Assay Kit, Thermo Fisher Scientific).

Each BALF sample was prepared in triplicate, except for one SEA affected horse (BALF 9) due to limited protein availability. 45 µg of total protein per sample were subjected to FASP using 10 kDa cut-off filters (Nanosep Omega, Pall Corporation, New York, USA). Proteins were retained on the FASP filter and washed at 14,000 x *g* with 0.1 M Tris-HCl pH 8.5. Alkylation was performed by incubation with 0.05 M iodoacetamide (Promega, Wisconsin, USA) for 20 min. After three washing steps with 0.1 M Tris-HCl, pH 8.0, at 14,000 x *g*, proteins were pre-digested for 4 h at 37 °C with 1 µg endoproteinase Lys-C (Mass Spec Grade, Promega, Wisconsin, USA) in 0.1 M Tris-HCl, pH 8.0. Next, digestion was continued by addition of 1 µg trypsin (Trypsin Gold Mass Spec Grade, Promega) in 0.05 M ammonium bicarbonate and incubated overnight at 37 °C. Tryptic peptides were recovered by centrifugation, acidified with 1% of formic acid (LC-MS grade, Thermo Fisher Scientific) and desalted using C18 Stage tips as described^92^.

### Quantitative proteome analysis by liquid chromatography-mass spectrometry (LC-MS)

Peptides were reconstituted in 15 µl of 0.05% trifluoroacetic acid (TFA) and 4% acetonitrile. One microliter of the solution was analyzed using an Ultimate 3000 reverse-phase capillary nano-liquid chromatography system connected to a Q Exactive HF mass spectrometer (Thermo Fisher Scientific).

Samples were injected and concentrated on a trap column (Acclaim PepMap100 C18, 3 μm, 100 Å, 75 μm i.d. x 2 cm, Thermo Fisher Scientific). After switching the trap column inline, liquid chromatography separations were performed on a capillary column (Acclaim PepMap100 C18, 2 μm, 100 Å, 75 μm i.d. x 50 cm, Thermo Fisher Scientific) at a flow rate of 300 nl/min. Mobile phase A contained 0.1% formic acid in water and mobile phase B contained 0.1% formic acid in 80% acetonitrile/20% water. The column was pre-equilibrated with 5% mobile phase B, followed by an increase of 5–44% mobile phase B over 35 min. Mass spectra were acquired in a data-dependent mode, utilizing a single MS survey scan (m/z 350–1650) at a resolution of 60,000 in the Orbitrap, followed by MS/MS scans of the 15 most intense precursor ions with a resolution of 15,000. The dynamic exclusion time was set to 20 s and automatic gain control was set to 3 × 10^6^ or 1 × 10^5^ for MS or MS/MS scans, respectively.

### Data analysis

MS and MS/MS raw data were analyzed using the MaxQuant software package (version 2.0.1.0) with the integrated Andromeda peptide search engine^93^. Data were searched against the *Equus caballus* reference proteome from Uniprot (69,389 proteins, Proteome ID UP000002281, downloaded July 7, 2023). The equine reference proteome was supplemented with a set of equine mucin amino acid sequences identified by BLAST searches of recently discovered human and bovine mucin sequences^94^ against the equine genome (GCF_002863925.1, EquCab3.0, supplementary file 2). Default parameters were used except for enabling the options ‘label-free quantification (LFQ)’ and ‘match between runs’.

Data were filtered using the software Perseus version 1.6.14^95^. LFQ values were log₂-transformed prior to analysis. Statistical analyses were performed and visualized using Perseus version 1.6.14, Python (v3.10, https://www.python.org/) with the pandas (v2.2.3)^96^, numpy (v2.2.3)^97^, scipy (v1.15.2)^98^, statsmodels (v0.14.4)^99^, matplotlib-venn (v1.1.2, https://github.com/konstantint/matplotlib-venn), scikit-learn (v1.5.1)^100^ and seaborn (v0.13.2)^101^ packages, R (v4.2, https://www.R-project.org/) with the dplyr (v 1.1.4, https://dplyr.tidyverse.org) and ggplot2 (v 3.5.1)^102^ packages and GraphPad Prism 10.

For comparisons of protein counts, PCA and heatmap analyses, mean log_2_ LFQ intensities from three technical replicates per BALF sample were used. Comparisons of protein counts between experimental groups (MEA, SEA or healthy) were performed using a one-way ANOVA following Shapiro–Wilk normality testing. A 3-set Venn diagram was used for the illustration of groupwise protein detection overlaps.

Principal component analysis (PCA) was conducted on log₂-transformed values after imputation in Perseus (normal distribution, width = 0.3, down shift = 1.8), and the first two components were visualized. For hierarchical clustering, one-way ANOVA was applied to log₂ LFQ intensities across SEA, MEA, and healthy controls, followed by Benjamini–Hochberg correction. The 100 proteins with the lowest adjusted p-values among significant results were z-score normalized per protein to emphasize relative abundance patterns and clustered using Euclidean distance and average linkage, with dendrograms calculated identically for both proteins (rows) and samples (columns). Pairwise group differences were evaluated using Tukey’s Honest Significant Difference test to determine the specific comparisons underlying the clustering patterns. For these protein counts, PCA and heatmap analyses, proteins were considered detected if quantified in ≥ 2 technical replicates of at least one BALF across all samples, or groupwise (MEA, SEA or healthy) if detected in ≥ 2 technical replicates of at least one BALF sample per group.

For mean log₂ fold changes in protein LFQ intensities between experimental groups (SEA vs. healthy, MEA vs. healthy, MEA vs. SEA), in the following designated as differential abundance analysis, and ROC curve and correlation analyses, only proteins that were identified with valid log₂ LFQ values in at least 50% within at least one experimental group were used for downstream analysis. Missing values were replaced from normal distribution (imputation) using the Perseus default settings (width 0.3, down shift 1.8).

Mean log₂ fold changes in protein LFQ intensities between experimental groups (SEA vs. healthy, MEA vs. healthy, MEA vs. SEA) were calculated using an unpaired two-tailed Student’s t-test in Perseus. A permutation-based false discovery rate (FDR) approach with S₀ = 0.1 was applied to yield-adjusted p-values (= q-values). Proteins with q-values ≤ 0.05, log_2_ fold changes ≤ −1 or ≥ 1 were considered statistically significant. Volcano plots were generated by plotting the − log₁₀ p-values against the mean log₂ fold changes of protein log₂ LFQ intensities.

To evaluate the discriminatory potential of each protein between healthy and asthmatic horses (SEA and MEA), we applied ROC curve analysis, which provides a threshold-independent measure of classification performance. The resulting AUC reflects the degree of separation between groups, with higher AUC values indicating stronger discriminatory ability and thus suitability for identifying candidate biomarkers. ROC curves and the corresponding AUCs were calculated for log₂-transformed LFQ intensities of each protein, comparing healthy horses to diseased animals (SEA and MEA combined). Proteins elevated in EA exceeding AUC thresholds of ≥ 0.8 were reported. Statistical uncertainty was assessed via stratified bootstrapping (1,000 resamples) to derive a 95% confidence interval.

Phenotype-specific ROC analyses were performed for SEA vs. MEA, SEA vs. healthy, and MEA vs. healthy comparisons. Proteins were classified as SEA- or MEA-associated if they showed high discrimination between SEA and MEA (AUC ≥ 0.8 in the respective direction) and between the associated phenotype and healthy controls (AUC ≥ 0.8), while showing limited discrimination of the other phenotype from healthy controls (AUC < 0.7).

To visualize the protein abundance distribution, a waterfall plot was generated, ranking proteins by the arithmetic mean of their log₂ LFQ intensities across all BALF samples regardless of group assignments.

To identify the least variable non differential abundant proteins, the median absolute deviation (MAD) was used as a robust metric of protein abundance variability across BALF samples, as it is less sensitive to outliers than standard deviation^103^. The top ten proteins without significant differences between groups (one-way ANOVA, *p* > 0.05) and the lowest MAD were reported.

Functional enrichment analyses were based on human Ensembl gene identifiers, as the limited functional annotation of the equine genome necessitated homolog mapping. All proteins identified in the differential abundance analyses (MEA vs. healthy and SEA vs. healthy) were mapped to human homologs using the g:Orth algorithm implemented in the g:Profiler webserver (https://biit.cs.ut.ee/gprofiler/convert, retrieved: 04/2025) with additional manual curation where necessary. Gene symbols were extracted and used for further analysis. Proteins were ranked as described by Locard–Paulet et al. (–log₁₀(p) × sign(log₂FC)) prior to analysis^104^. For the ranked g:Profiler analysis, only significantly increased proteins with a fold change ≥ 2 were analyzed as multiquery with default parameters. Additionally, GSEA was performed in pre-ranked mode, also including decreased proteins regardless of fold chance thresholds using GSEA software (Broad Institute, v4.3.2 https://software.broadinstitute.org) with the Hallmark gene set collection (MSigDB v2024.1, h.all.v2024.1.Hs.symbols.gmt), filtered to gene sets sized 15–500 genes. Gene symbols were used as input and only positively enriched sets were considered. Significance was defined as FDR q < 0.25.

For the correlation of proteomic with cytological data, Spearman’s rank correlation coefficients (ρ) were calculated between mean log₂-transformed LFQ intensities of triplicates per BALF sample and the corresponding percentage of neutrophils to explore associations between protein abundance and neutrophil granulocyte percentages. The ten proteins with the highest positive absolute ρ were identified.

### Immunohistochemistry

Formalin-fixed, paraffin-embedded (FFPE) lung tissue of healthy horses and horses with SEA (*n* = 4 per group), previously included in a study with an SEA diagnosis based on the clinical history and histologic lesions^34^ were cut at 3 μm thickness, mounted on adhesive glass slides, and dewaxed. Endogenous peroxidase was blocked by 0.5% H_2_O_2_. Antigen retrieval was performed using microwave heating in 10 mM citric acid, pH 6.0, containing 0.05% Triton X-100. Slides were blocked with 10% Roti-ImmunoBlock and 20% goat serum in PBS, incubated overnight at 4 °C with mouse monoclonal anti-MUC4 antibody (Abcam 60720, 1:1000) or rabbit polyclonal anti-PIGR antibody (ATLAS Antibodies HPA012012, 1:500). Since both antibodies were generated against human proteins, epitope-homologies between human and equine homologs were assessed first. The resulting E-values were below 10^− 5^, indicating sufficient similarity to support cross-reactivity^105^. Following repeated washes, slides were incubated with a secondary goat anti-mouse- or goat anti-rabbit-IgG antibody (BP-9200 and BA-1000, Vector Laboratories, Burlingame, California, USA), respectively, at a dilution of 1:200 for 30 min at room temperature. Color was developed by incubation in ABC solution (Vectastain Elite ABC-HRP Kit; Vector Laboratories) for 30 min, followed by repeated washes in PBS and exposure to diaminobenzidine. Slides were counterstained with hematoxylin.

### Utilization of large Language models

During the preparation of the manuscript, AI tools (DeepL [www.deepl.com], ChatGPT-4o [https://chatgpt.com], and DeepSeek-V3 [https://www.deepseek.com]) were used to support language and grammar checks, as well as the development of Python and R scripts.

### Ethics statement

All examinations and sampling of asthmatic horses were part of a routine clinical diagnostic respiratory disease workup for the benefit of the patients at the patient owner’s request. All examinations and sampling procedures involving healthy horses were part of the clinical training in veterinary education or of another study. All procedures were ethically approved by official authorities (State Office of Health and Social Affairs Berlin, Germany, StN 002/22, L 0269/18, G 0156/21 and Landesdirektion Sachsen, Germany, TVV22/20 file number 25–5131/490/23).

## Supplementary Information

Below is the link to the electronic supplementary material.

Supplementary Material 1

Supplementary Material 2

Supplementary Material 3

Supplementary Material 4

Supplementary Material 5

Supplementary Material 6

Supplementary Material 7

## Data Availability

The mass spectrometry proteomics data have been deposited to the ProteomeXchange Consortium via the PRIDE (Proteomics IDEntifications, https://www.ebi.ac.uk/pride/)^106^ partner repository. The dataset can be accessed using the identifier PXD070776. All code used for data processing and analysis is available from the corresponding author (LM) upon request.

## References

[1] Couetil, L. L. et al. Inflammatory airway disease of horses–revised consensus statement. *J. Vet. Intern. Med.* **30**, 503–515. 10.1111/jvim.13824 (2016). 26806374 10.1111/jvim.13824 PMC4913592

[2] Wysocka, B. & Kluciński, W. Usefulness of the assessment of discharge accumulation in the lower airways and tracheal septum thickening in the differential diagnosis of recurrent airway obstruction (RAO) and inflammatory airway disease (IAD) in the horse. *Pol. J. Vet. Sci.* **17**, 247–253. 10.2478/pjvs-2014-0035 (2014). 24988850 10.2478/pjvs-2014-0035

[3] Drespling, J. et al. Endoscopically assessed mucus parameters in equine asthma: relationship to clinical history and cytological findings data. *Equine Veterinary Journal* https://doi.org/10.1111/evj.70002 10.1111/evj.70002 PMC13041601 40704584

[4] Maxie, G. *Jubb, Kennedy & Palmer’s pathology of domestic animals: volume 2*. Vol. 2Elsevier health sciences, (2015).

[5] Larsen, M. et al. Associations between clinical signs, endoscopic and cytological findings in equine Bronchoalveolar lavage samples. *Equine Veterinary Education*10.1111/eve.14174

[6] Ferrari, C. R. et al. Horses with pasture asthma have airway remodeling that is characteristic of human asthma. *Vet. Pathol.* **55**, 144–158. 10.1177/0300985817741729 (2018). 29254472 10.1177/0300985817741729

[7] Williams, K. & Roman, J. Studying human respiratory disease in animals–role of induced and naturally occurring models. *J. Pathol.* **238**, 220–232. 10.1002/path.4658 (2016). 26467890 10.1002/path.4658

[8] Léguillette, R. Recurrent airway obstruction—heaves. *Veterinary Clinics: Equine Pract.* **19**, 63–86. 10.1016/S0749-0739(02)00067-6 (2003). 10.1016/s0749-0739(02)00067-6 12747662

[9] Bullone, M. & Lavoie, J. P. The equine asthma model of airway remodeling: from a veterinary to a human perspective. *Cell Tissue Res.* **380**, 223–236. 10.1007/s00441-019-03117-4 (2020). 31713728 10.1007/s00441-019-03117-4

[10] Woodrow, J. S., Sheats, M. K., Cooper, B., Bayless, R. & Asthma The use of animal models and their translational utility. *Cells* **12**, 1091. 10.3390/cells12071091 (2023). 37048164 10.3390/cells12071091 PMC10093022

[11] Bullone, M. & Lavoie, J. P. Asthma of horses and men--how can equine heaves help Us better understand human asthma immunopathology and its functional consequences? *Mol. Immunol.* **66**, 97–105. 10.1016/j.molimm.2014.12.005 (2015). 25547716 10.1016/j.molimm.2014.12.005

[12] Leduc, L., Leclère, M. & Lavoie, J. P. Towards personalized medicine for the treatment of equine asthma. *Vet. J.* **305**, 106125. 10.1016/j.tvjl.2024.106125 (2024). 38704018 10.1016/j.tvjl.2024.106125

[13] Fahy, J. V. & Dickey, B. F. Airway mucus function and dysfunction. *N Engl. J. Med.* **363**, 2233–2247. 10.1056/NEJMra0910061 (2010). 21121836 10.1056/NEJMra0910061 PMC4048736

[14] Hill, D. B., Button, B., Rubinstein, M. & Boucher, R. C. Physiology and pathophysiology of human airway mucus. *Physiol. Rev.* **102**, 1757–1836. 10.1152/physrev.00004.2021 (2022). 35001665 10.1152/physrev.00004.2021 PMC9665957

[15] Bansil, R. & Turner, B. S. The biology of mucus: Composition, synthesis and organization. *Adv. Drug Deliv. Rev.* **124**, 3–15. 10.1016/j.addr.2017.09.023 (2018). 28970050 10.1016/j.addr.2017.09.023

[16] Jefcoat, A. M. et al. Persistent mucin glycoprotein alterations in equine recurrent airway obstruction. *Am. J. Physiol. Lung Cell. Mol. Physiol.* **281**, L704–712. 10.1152/ajplung.2001.281.3.L704 (2001). 11504699 10.1152/ajplung.2001.281.3.L704

[17] Gerber, V., King, M., Schneider, D. A. & Robinson, N. E. Tracheobronchial mucus viscoelasticity during environmental challenge in horses with recurrent airway obstruction. *Equine Vet. J.* **32**, 411–417. 10.2746/042516400777591183 (2000). 11037263 10.2746/042516400777591183

[18] Rousseau, K. et al. Muc5b and Muc5ac are the major oligomeric mucins in equine airway mucus. *Am. J. Physiol. Lung Cell. Mol. Physiol.* **292**, L1396–1404. 10.1152/ajplung.00444.2006 (2007). 17293373 10.1152/ajplung.00444.2006

[19] Rousseau, K. et al. Muc5b is the major polymeric mucin in mucus from thoroughbred horses with and without airway mucus accumulation. *PLoS One*. **6**, e19678. 10.1371/journal.pone.0019678 (2011). 21602926 10.1371/journal.pone.0019678 PMC3094342

[20] Gerber, V. et al. Mucin genes in horse airways: MUC5AC, but not MUC2, May play a role in recurrent airway obstruction. *Equine Vet. J.* **35**, 252–257. 10.2746/042516403776148291 (2003). 12755427 10.2746/042516403776148291

[21] Bright, L. et al. Functional modelling of an equine Bronchoalveolar lavage fluid proteome provides experimental confirmation and functional annotation of equine genome sequences. *Anim. Genet.* **42**, 395–405. 10.1111/j.1365-2052.2010.02158.x (2011). 21749422 10.1111/j.1365-2052.2010.02158.x

[22] Bright, L. A. et al. Modeling the pasture-associated severe equine asthma Bronchoalveolar lavage fluid proteome identifies molecular events mediating neutrophilic airway inflammation. *Veterinary Medicine: Res. Rep.* **10**, 43–63. 10.2147/VMRR.S194427 (2019). 10.2147/VMRR.S194427 PMC6504673 31119093

[23] Feutz, M. M. et al. Proteomic analysis of Bronchoalveolar lavage fluid in an equine model of asthma during a natural antigen exposure trial. *J. Integr. OMICS*. **2**, 123–131. 10.5584/jiomics.v2i2.112 (2012).

[24] Racine, J. et al. Comparison of genomic and proteomic data in recurrent airway obstruction affected horses using ingenuity pathway analysis^®^. *BMC Vet. Res.* **7**, 1–10. 10.1186/1746-6148-7-48 (2011). 10.1186/1746-6148-7-48 PMC3174119 21843342

[25] Mandrekar, J. N. Receiver operating characteristic curve in diagnostic test assessment. *J. Thorac. Oncol.* **5**, 1315–1316. 10.1097/JTO.0b013e3181ec173d (2010). 20736804 10.1097/JTO.0b013e3181ec173d

[26] Cohen, J. *Statistical Power Analysis for the Behavioral Sciences* 2nd edn, Vol. 567 (Routledge, 2013).

[27] Couetil, L. et al. Equine asthma: current Understanding and future directions. *Front. Vet. Sci.* **7**, 450. 10.3389/fvets.2020.00450 (2020). 32903600 10.3389/fvets.2020.00450 PMC7438831

[28] Ward, C. et al. Airway inflammation, basement membrane thickening and bronchial hyperresponsiveness in asthma. *Thorax* **57**, 309–316. 10.1136/thorax.57.4.309 (2002). 11923548 10.1136/thorax.57.4.309 PMC1746305

[29] Woodruff, P. G. et al. T-helper type 2–driven inflammation defines major subphenotypes of asthma. *Am. J. Respir. Crit Care Med.* **180**, 388–395. 10.1164/rccm.200903-0392OC (2009). 19483109 10.1164/rccm.200903-0392OC PMC2742757

[30] Setlakwe, E. L., Lemos, K. R., Lavoie-Lamoureux, A., Duguay, J. D. & Lavoie, J. P. Airway collagen and elastic fiber content correlates with lung function in equine heaves. *Am. J. Physiol. Lung Cell. Mol. Physiol.* **307**, L252–260. 10.1152/ajplung.00019.2014 (2014). 24879055 10.1152/ajplung.00019.2014

[31] Bullone, M., Chevigny, M., Allano, M., Martin, J. G. & Lavoie, J. P. Technical and physiological determinants of airway smooth muscle mass in endobronchial biopsy samples of asthmatic horses. *J. Appl. Physiol. (1985)*. **117**, 806–815. 10.1152/japplphysiol.00468.2014 (2014). 25103978 10.1152/japplphysiol.00468.2014

[32] Herszberg, B., Ramos-Barbón, D., Tamaoka, M., Martin, J. G. & Lavoie, J. P. Heaves, an asthma-like equine disease, involves airway smooth muscle remodeling. *J. Allergy Clin. Immunol.* **118**, 382–388. 10.1016/j.jaci.2006.03.044 (2006). 16890762 10.1016/j.jaci.2006.03.044

[33] Leclere, M. et al. Effect of antigenic exposure on airway smooth muscle remodeling in an equine model of chronic asthma. *Am. J. Respir Cell. Mol. Biol.* **45**, 181–187. 10.1165/rcmb.2010-0300OC (2011). 20935189 10.1165/rcmb.2010-0300OC

[34] Range, F., Mundhenk, L. & Gruber, A. D. A soluble secreted glycoprotein (eCLCA1) is overexpressed due to goblet cell hyperplasia and metaplasia in horses with recurrent airway obstruction. *Vet. Pathol.* **44**, 901–911. 10.1354/vp.44-6-901 (2007). 18039903 10.1354/vp.44-6-901

[35] Lugo, J. et al. Airway inflammation is associated with mucous cell metaplasia and increased intraepithelial stored mucosubstances in horses. *Vet. J.* **172**, 293–301. 10.1016/j.tvjl.2005.04.018 (2006). 15925524 10.1016/j.tvjl.2005.04.018

[36] Bessonnat, A., Hélie, P., Grimes, C. & Lavoie, J. P. Airway remodeling in horses with mild and moderate asthma. *J. Vet. Intern. Med.* **36**, 285–291. 10.1111/jvim.16333 (2022). 34877706 10.1111/jvim.16333 PMC8783337

[37] ORDOÑEZ, C. L. et al. Mild and moderate asthma is associated with airway goblet cell hyperplasia and abnormalities in mucin gene expression. *Am. J. Respir. Crit Care Med.* **163**, 517–523. 10.1164/ajrccm.163.2.2004039 (2001). 11179133 10.1164/ajrccm.163.2.2004039

[38] Tan, H. T. T. et al. Tight junction, mucin, and inflammasome-related molecules are differentially expressed in eosinophilic, mixed, and neutrophilic experimental asthma in mice. *Allergy* **74**, 294–307. 10.1111/all.13619 (2019). 30267575 10.1111/all.13619

[39] Evans, C. M. et al. The polymeric mucin Muc5ac is required for allergic airway hyperreactivity. *Nat. Commun.* **6**, 6281. 10.1038/ncomms7281 (2015). 25687754 10.1038/ncomms7281 PMC4333679

[40] Tajiri, T. et al. Pathophysiological relevance of sputum MUC5AC and MUC5B levels in patients with mild asthma. *Allergology Int.* **71**, 193–199. 10.1016/j.alit.2021.09.003 (2022). 10.1016/j.alit.2021.09.003 34656442

[41] Lachowicz-Scroggins, M. E. et al. Abnormalities in MUC5AC and MUC5B protein in airway mucus in asthma. *Am. J. Respir. Crit Care Med.* **194**, 1296–1299. 10.1164/rccm.201603-0526LE (2016). 27845589 10.1164/rccm.201603-0526LE PMC5114443

[42] Nath, S. & Mukherjee, P. MUC1: a multifaceted oncoprotein with a key role in cancer progression. *Trends Mol. Med.* **20**, 332–342. 10.1016/j.molmed.2014.02.007 (2014). 24667139 10.1016/j.molmed.2014.02.007 PMC5500204

[43] Chaturvedi, P., Singh, A. P. & Batra, S. K. Structure, evolution, and biology of the MUC4 mucin. *Faseb j.* **22**, 966–981. 10.1096/fj.07-9673rev (2008). 18024835 10.1096/fj.07-9673rev PMC2835492

[44] Soto, P., Zhang, J. & Carraway, K. L. Enzymatic cleavage as a processing step in the maturation of Muc4/sialomucin complex. *J. Cell. Biochem.* **97**, 1267–1274. 10.1002/jcb.20718 (2006). 16329125 10.1002/jcb.20718

[45] Kato, K., Lillehoj, E. P., Lu, W. & Kim, K. C. MUC1: the first respiratory mucin with an anti-inflammatory function. *J. Clin. Med.* **6**10.3390/jcm6120110 (2017). 10.3390/jcm6120110 PMC5742799 29186029

[46] Blalock, T. D., Spurr-Michaud, S. J., Tisdale, A. S. & Gipson, I. K. Release of membrane-associated mucins from ocular surface epithelia. *Invest. Ophthalmol. Vis. Sci.* **49**, 1864–1871. 10.1167/iovs.07-1081 (2008). 18436821 10.1167/iovs.07-1081 PMC2622730

[47] Joo, N. S. et al. Proteomic analysis of pure human airway gland mucus reveals a large component of protective proteins. *PLOS ONE*. **10**, e0116756. 10.1371/journal.pone.0116756 (2015). 25706550 10.1371/journal.pone.0116756 PMC4338240

[48] Damera, G., Xia, B. & Sachdev, G. P. IL-4 induced MUC4 enhancement in respiratory epithelial cells in vitro is mediated through JAK-3 selective signaling. *Respir. Res.* **7**, 1–12. 10.1186/1465-9921-7-39 (2006). 16551361 10.1186/1465-9921-7-39 PMC1435893

[49] Zhou, X. et al. Sialylation of MUC4β N-glycans by ST6GAL1 orchestrates human airway epithelial cell differentiation associated with type-2 inflammation. *JCI Insight*. **4**10.1172/jci.insight.122475 (2019). 10.1172/jci.insight.122475 PMC6483602 30730306

[50] Hattori, T., Zhou, X., Trudeau, J. B. & Wenzel, S. E. MUC4 protein is increased in severe asthmatic bronchial epithelial cells. *Am. J. Respir. Crit. Care Med. ***187**, A2404

[51] Padoan, E. et al. Gene expression profiles of the Immuno-Transcriptome in equine asthma. *Animals* **13**, 4. 10.3390/ani13010004 (2023). 10.3390/ani13010004 PMC9817691 36611613

[52] Fahy, J. V. Goblet cell and mucin gene abnormalities in Asthma*. *Chest* **122**, 320S–326S. 10.1378/chest.122.6_suppl.320S (2002). 12475809 10.1378/chest.122.6_suppl.320s

[53] Gorman, H., Moreau, F., Dufour, A. & Chadee, K. IgGFc-binding protein and MUC2 mucin produced by colonic goblet-like cells spatially interact non-covalently and regulate wound healing. *Front. Immunol.* 14–2023. 10.3389/fimmu.2023.1211336 (2023). 10.3389/fimmu.2023.1211336 PMC10285406 37359538

[54] Gorman, H., Moreau, F., Kim, A. & Chadee, K. FCGBP stabilizes colonic MUC2 mucin structural integrity in innate host defense against entamoeba histolytica. *FASEB J.* **35**10.1096/fasebj.2021.35.S1.00451 (2021).

[55] Ehrencrona, E. et al. The IgGFc-binding protein FCGBP is secreted with all GDPH sequences cleaved but maintained by interfragment disulfide bonds. *J. Biol. Chem.* **297**10.1016/j.jbc.2021.100871 (2021). 10.1016/j.jbc.2021.100871 PMC8267560 34126068

[56] Park, S. W. et al. The protein disulfide isomerase AGR2 is essential for production of intestinal mucus. *Proc. Natl. Acad. Sci. U S A*. **106**, 6950–6955. 10.1073/pnas.0808722106 (2009). 19359471 10.1073/pnas.0808722106 PMC2678445

[57] Schroeder, B. W. et al. AGR2 is induced in asthma and promotes allergen-induced mucin overproduction. *Am. J. Respir Cell. Mol. Biol.* **47**, 178–185. 10.1165/rcmb.2011-0421OC (2012). 22403803 10.1165/rcmb.2011-0421OC PMC3423459

[58] Varki, A., Cummings, R. D., Esko, J. D. et al. Essentials of Glycobiology [Internet]. 3rd edn. Cold Spring Harbor Laboratory Press (2015–2017). https://www.ncbi.nlm.nih.gov/books/NBK310274/. 27010055

[59] Bennett, E. P. et al. Control of mucin-type O-glycosylation: A classification of the polypeptide GalNAc-transferase gene family. *Glycobiology* **22**, 736–756. 10.1093/glycob/cwr182 (2011). 22183981 10.1093/glycob/cwr182 PMC3409716

[60] Venkitachalam, S. et al. Biochemical and functional characterization of glycosylation-associated mutational landscapes in colon cancer. *Sci. Rep.* **6**, 23642. 10.1038/srep23642 (2016). 27004849 10.1038/srep23642 PMC4804330

[61] Solatycka, A. et al. MUC1 in human and murine mammary carcinoma cells decreases the expression of core 2 β1,6-N-acetylglucosaminyltransferase and β-galactoside α2,3-sialyltransferase. *Glycobiology* **22**, 1042–1054. 10.1093/glycob/cws075 (2012). 22534569 10.1093/glycob/cws075

[62] Xia, B., Royall, J. A., Damera, G., Sachdev, G. P. & Cummings, R. D. Altered O-glycosylation and sulfation of airway mucins associated with cystic fibrosis. *Glycobiology* **15**, 747–775. 10.1093/glycob/cwi061 (2005). 15994837 10.1093/glycob/cwi061

[63] Harris, E. S. et al. Reduced sialylation of airway mucin impairs mucus transport by altering the biophysical properties of mucin. *Sci. Rep.* **14**, 16568. 10.1038/s41598-024-66510-2 (2024). 39019950 10.1038/s41598-024-66510-2 PMC11255327

[64] Crouzier, T. et al. Modulating mucin hydration and lubrication by deglycosylation and polyethylene glycol binding. *Adv. Mater. Interfaces*. **2**, 1500308. 10.1002/admi.201500308 (2015).

[65] Kameyama, A., Nishijima, R. & Yamakoshi, K. Bmi-1 regulates mucin levels and mucin O-glycosylation in the submandibular gland of mice. *PLOS ONE*. **16**, e0245607. 10.1371/journal.pone.0245607 (2021). 33465144 10.1371/journal.pone.0245607 PMC7815129

[66] Jenssen, A. O., Olav, S., Harbitz, O. & and The importance of lysozyme for the viscosity of sputum from patients with chronic obstructive lung disease. *Scand. J. Clin. Lab. Investig.* **40**, 727–731. 10.3109/00365518009095588 (1980). 7280551 10.3109/00365518009095588

[67] Ablimit, A. et al. Changes in water channel Aquaporin 1 and Aquaporin 5 in the small airways and the alveoli in a rat asthma model. *Micron* **45**, 68–73. 10.1016/j.micron.2012.10.016 (2013). 23199524 10.1016/j.micron.2012.10.016

[68] Verkman, A. S. Role of Aquaporins in lung liquid physiology. *Respir Physiol. Neurobiol.* **159**, 324–330. 10.1016/j.resp.2007.02.012 (2007). 17369110 10.1016/j.resp.2007.02.012 PMC3315286

[69] Erickson, N. A., Gruber, A. D. & Mundhenk, L. The family of chloride channel Regulator, Calcium-activated proteins in the feline respiratory tract: A comparative perspective on airway diseases in man and animal models. *J. Comp. Pathol.* **174**, 39–53. 10.1016/j.jcpa.2019.10.193 (2020). 31955802 10.1016/j.jcpa.2019.10.193

[70] Fernandez-Blanco, J. A. et al. Attached stratified mucus separates bacteria from the epithelial cells in COPD lungs. *JCI Insight*. **3**10.1172/jci.insight.120994 (2018). 10.1172/jci.insight.120994 PMC6171804 30185674

[71] Plog, S., Mundhenk, L., Klymiuk, N. & Gruber, A. D. Genomic, tissue expression, and protein characterization of pCLCA1, a putative modulator of cystic fibrosis in the pig. *J. Histochem. Cytochem.* **57**, 1169–1181. 10.1369/jhc.2009.954594 (2009). 19755716 10.1369/jhc.2009.954594 PMC2778090

[72] Leverkoehne, I. & Gruber, A. D. The murine mCLCA3 (Alias gob-5) protein is located in the mucin granule membranes of Intestinal, Respiratory, and uterine goblet cells. *J. Histochem. Cytochemistry*. **50**, 829–838. 10.1177/002215540205000609 (2002). 10.1177/002215540205000609 12019299

[73] Anton, F., Leverkoehne, I., Mundhenk, L., Thoreson, W. B. & Gruber, A. D. Overexpression of eCLCA1 in small airways of horses with recurrent airway obstruction. *J. Histochem. Cytochem.* **53**, 1011–1021. 10.1369/jhc.4A6599.2005 (2005). 15879574 10.1369/jhc.4A6599.2005 PMC1383431

[74] Lycke, N., Erlandsson, L., Ekman, L., Schön, K. & Leanderson, T. Lack of J chain inhibits the transport of gut IgA and abrogates the development of intestinal antitoxic protection. *J. Immunol.* **163**, 913–919. 10.4049/jimmunol.163.2.913 (1999). 10395687

[75] Kawasaki, K., Ohta, Y., Castro, C. D. & Flajnik, M. F. The Immunoglobulin J chain is an evolutionarily co-opted chemokine. *Proc. Natl. Acad. Sci. U S A*. **121**, e2318995121. 10.1073/pnas.2318995121 (2024). 38215184 10.1073/pnas.2318995121 PMC10801876

[76] Mostov, K. E. Transepithelial transport of Immunoglobulins. *Annu. Rev. Immunol.* **12**, 63–84. 10.1146/annurev.iy.12.040194.000431 (1994). 8011293 10.1146/annurev.iy.12.040194.000431

[77] Wei, H. & Wang, J. Y. Role of polymeric Immunoglobulin receptor in IgA and IgM transcytosis. *Int. J. Mol. Sci.* **22**10.3390/ijms22052284 (2021). 10.3390/ijms22052284 PMC7956327 33668983

[78] Jentsch, M. C. et al. Aspergillus fumigatus binding IgA and IgG1 are increased in Bronchoalveolar lavage fluid of horses with neutrophilic asthma. *Front. Immunol.* **15–2024**10.3389/fimmu.2024.1406794 (2024). 10.3389/fimmu.2024.1406794 PMC11215007 38953030

[79] Robinson, N. E. et al. Coughing, mucus accumulation, airway obstruction, and airway inflammation in control horses and horses affected with recurrent airway obstruction. *Am. J. Vet. Res.* **64**, 550–557. 10.2460/ajvr.2003.64.550 (2003). 12755293 10.2460/ajvr.2003.64.550

[80] Richard, E. A., Fortier, G. D., Lekeux, P. M. & Van Erck, E. Laboratory findings in respiratory fluids of the poorly-performing horse. *Vet. J.* **185**, 115–122. 10.1016/j.tvjl.2009.05.003 (2010). 19481964 10.1016/j.tvjl.2009.05.003

[81] Gerber, V., Ii, S., Robinson, N. & H. & Owner assessment in judging the efficacy of airway disease treatment. *Equine Vet. J.* **43**, 153–158. 10.1111/j.2042-3306.2010.00156.x (2011). 21592208 10.1111/j.2042-3306.2010.00156.x

[82] Fogarty, U. & Buckley, T. Bronchoalveolar lavage findings in horses with exercise intolerance. *Equine Vet. J.* **23**, 434–437. 10.1111/j.2042-3306.1991.tb03756.x (1991). 1778160 10.1111/j.2042-3306.1991.tb03756.x

[83] Couetil, L. & Denicola, D. Blood gas, plasma lactate and Bronchoalveolar lavage cytology analyses in racehorses with respiratory disease. *Equine Vet. J.* **31**, 77–82. 10.1111/j.2042-3306.1999.tb05193.x (1999). 10.1111/j.2042-3306.1999.tb05193.x 10659227

[84] Wasko, A. et al. Evaluation of a risk-screening questionnaire to detect equine lung inflammation: results of a large field study. *Equine Vet. J.* **43**, 145–152. 10.1111/j.2042-3306.2010.00150.x (2011). 21592207 10.1111/j.2042-3306.2010.00150.x

[85] Beekman, L., Tohver, T. & Léguillette, R. Comparison of cytokine mRNA expression in the Bronchoalveolar lavage fluid of horses with inflammatory airway disease and Bronchoalveolar lavage mastocytosis or neutrophilia using REST software analysis. *J. Vet. Intern. Med.* **26**, 153–161. 10.1111/j.1939-1676.2011.00847.x (2012). 22168153 10.1111/j.1939-1676.2011.00847.x

[86] Hughes, K. J. et al. Evaluation of cytokine mRNA expression in Bronchoalveolar lavage cells from horses with inflammatory airway disease. *Vet. Immunol. Immunopathol.* **140**, 82–89. 10.1016/j.vetimm.2010.11.018 (2011). 21194756 10.1016/j.vetimm.2010.11.018

[87] Hoffman, A., Mazan, M. & Ellenberg, S. Association between Bronchoalveolar lavage cytologic features and airway reactivity in horses with a history of exercise intolerance. *Am. J. Vet. Res.* **59**, 176–181. 10.2460/ajvr.1998.59.02.176 (1998). 9492932

[88] Couëtil, L. L., Rosenthal, F. S., DeNicola, D. B. & Chilcoat, C. D. Clinical signs, evaluation of Bronchoalveolar lavage fluid, and assessment of pulmonary function in horses with inflammatory respiratory disease. *Am. J. Vet. Res.* **62**, 538–546. 10.2460/ajvr.2001.62.538 (2001). 11327461 10.2460/ajvr.2001.62.538

[89] Derksen, F. J., Scott, J. S., Miller, D. C., Slocombe, R. F. & Robinson, N. E. Bronchoalveolar lavage in ponies with recurrent airway obstruction (heaves). *Am. Rev. Respir. Dis.* **132**, 1066–1070. 10.1164/arrd.1985.132.5.1066 (1985). 4062037 10.1164/arrd.1985.132.5.1066

[90] Hare, J. E. & Viel, L. Pulmonary eosinophilia associated with increased airway responsiveness in young racing horses. *J. Vet. Intern. Med.* **12**, 163–170. 10.1111/j.1939-1676.1998.tb02112.x (1998). 9595377 10.1111/j.1939-1676.1998.tb02112.x

[91] Mundhenk, L., Bartenschlager, F., Gruber, A., Gehlen, H., Weise, C., Kuropka, B., Dumke, F. L., Biomarkers for Diagnosing Equine Asthma. Patent: EP4260906 (2023).

[92] Rappsilber, J., Mann, M. & Ishihama, Y. Protocol for micro-purification, enrichment, pre-fractionation and storage of peptides for proteomics using stagetips. *Nat. Protoc.* **2**, 1896–1906. 10.1038/nprot.2007.261 (2007). 17703201 10.1038/nprot.2007.261

[93] Tyanova, S., Temu, T. & Cox, J. The MaxQuant computational platform for mass spectrometry-based shotgun proteomics. *Nat. Protoc.* **11**, 2301–2319. 10.1038/nprot.2016.136 (2016). 27809316 10.1038/nprot.2016.136

[94] Pajic, P. et al. A mechanism of gene evolution generating mucin function. *Sci. Adv.* **8**, eabm8757. 10.1126/sciadv.abm8757 (2022). 36026444 10.1126/sciadv.abm8757 PMC9417175

[95] Tyanova, S. et al. The perseus computational platform for comprehensive analysis of (prote)omics data. *Nat. Methods*. **13**, 731–740. 10.1038/nmeth.3901 (2016). 27348712 10.1038/nmeth.3901

[96] McKinney, W. Data structures for statistical computing in python. *SciPy* **445** (51-56). 10.25080/Majora-92bf1922-00a (2010).

[97] Harris, C. R. et al. Array programming with numpy. *Nature* **585**, 357–362. 10.1038/s41586-020-2649-2 (2020). 32939066 10.1038/s41586-020-2649-2 PMC7759461

[98] Virtanen, P. et al. SciPy 1.0: fundamental algorithms for scientific computing in python. *Nat. Methods*. **17**, 261–272. 10.1038/s41592-019-0686-2 (2020). 32015543 10.1038/s41592-019-0686-2 PMC7056644

[99] Seabold, S. & Perktold, J. Statsmodels: econometric and statistical modeling with python. *SciPy* **7**, 92–96. 10.25080/Majora-92bf1922-011 (2010).

[100] Pedregosa, F. et al. Scikit-learn: machine learning in python. *J. Mach. Learn. Res.* **12**, 2825–2830. 10.48550/arXiv.1201.0490 (2011).

[101] Waskom, M. L. Seaborn: statistical data visualization. *J. Open. Source Softw.* **6**, 3021. 10.21105/joss.03021 (2021).

[102] Wickham, H. & Sievert, C. *ggplot2: Elegant Graphics for Data Analysis* Vol. 10 (Springer, 2009).

[103] Leys, C., Ley, C., Klein, O., Bernard, P. & Licata, L. Detecting outliers: do not use standard deviation around the mean, use absolute deviation around the median. *J. Exp. Soc. Psychol.* **49**, 764–766. 10.1016/j.jesp.2013.03.013 (2013).

[104] Locard-Paulet, M., Doncheva, N. T., Morris, J. H. & Jensen, L. J. Functional analysis of MS-Based proteomics data: from protein groups to networks. *Mol. Cell. Proteom.* **23**10.1016/j.mcpro.2024.100871 (2024). 10.1016/j.mcpro.2024.100871 PMC11667155 39486590

[105] McClain, S. Bioinformatic screening and detection of allergen cross-reactive IgE-binding epitopes. *Mol. Nutr. Food Res.* **61**, 1600676. 10.1002/mnfr.201600676 (2017). 28191711 10.1002/mnfr.201600676 PMC5573986

[106] Perez-Riverol, Y. et al. The PRIDE database at 20 years: 2025 update. *Nucleic Acids Res.* **53**10.1093/nar/gkae1011 (2025). D543-D553. 10.1093/nar/gkae1011 PMC11701690 39494541

